# The Impact of Accumulated Mutations in SARS-CoV-2 Variants on the qPCR Detection Efficiency

**DOI:** 10.3389/fcimb.2022.823306

**Published:** 2022-01-28

**Authors:** Liu Cao, Tiefeng Xu, Xue Liu, Yanxi Ji, Siyao Huang, Hong Peng, Chunmei Li, Deyin Guo

**Affiliations:** Centre for Infection and Immunity Study (CIIS), School of Medicine, Sun Yat-sen University, Shenzhen, China

**Keywords:** SARS-CoV-2, SARS-COV-2 variants, delta variant, quantitative PCR, qPCR detection efficiency

## Abstract

SARS-CoV-2 is evolving with mutations throughout the genome all the time and a number of major variants emerged, including several variants of concern (VOC), such as Delta and Omicron variants. In this study, we demonstrated that mutations in the regions corresponding to the sequences of the probes and 3’-end of primers have a significant impact on qPCR detection efficiency. We also found that the G28916T mutation of the N gene accounts for 78.78% sequenced genomes of Delta variant. It was found that detection sensitivity of G28916T mutant was 2.35 and 1.74 times less than that of the wt sequence and detection limit was reduced from 1 copy/μl to 10 copies/μl for the commercially available CP3 and CP4 primer/probe sets. These results indicate that the detection probes and primers should be optimized to keep maximal detection efficiency in response to the emergence of new variants.

## Introduction

Coronavirus disease 2019 (COVID-19) caused by severe acute respiratory syndrome coronavirus 2 (SARS-CoV-2) has caused a severe global pandemic. Virus detection is the first step to fight against COVID-19 epidemic. SARS-CoV-2 tests can be grouped as nucleic acid, serological, antigen, and ancillary tests, all of which play distinct roles in hospital, point-of-care, or large-scale population testing (Ravi et al., 2020; Shi et al., 2020). Especially, the real-time quantitative PCR (qPCR) method is the most common and the most widely used. qPCR-based assays performed on respiratory specimens have emerged as the cornerstone of COVID-19 diagnostic and screening testing (Corman et al., 2020; Vogels et al., 2020). However, mutations and deletions on the SARS-CoV-2 genome may reduce the detection efficiency of qPCR (Penarrubia et al., 2020; Ziegler et al., 2020; Hasan et al., 2021). Although the coronavirus has a certain proofreading ability (Robson et al., 2020), it still has a high mutation rate, which leads to the emergence of new variants. At present, the WHO has classified many variants of the SARS-CoV-2 (WHO, 2021), including the variants of concern (VOC), such as the Alpha variant (B.1.1.7), Beta variant (B.1.351), Gamma variant (P.1), Delta variant (B.1.671.2) and Omicron variant (B.1.1.529). Delta and Omicron variants are highly contagious, and have rapidly become the prevalent variants, accounting for more than 99% of SARS-CoV-2 variants from December 1 to December 27, 2021. Delta variant may lead to vaccine breakthrough infections associated with higher viral load and long duration of shedding (Reardon, 2021). Omicron variant has higher transmission capacity and exhibits stronger ability to evade the protection of previously existing antibodies and currently available vaccines (Cao et al., 2021; Cele et al., 2021; Chen et al., 2021; Liu et al., 2021; Planas et al., 2021). In this study, we systematically analysed the mutations of the SARS-CoV-2 genome and showed that some mutations that correspond to the target sequences of qPCR probe/primer may have significant impact on the detection efficiency.

## Methods

### Sources and Analyze of Sequences

As of 1 January to 19 February, 2021, there were a total of 89791 high-quality SARS-CoV-2 genomes sequences available in Global Initiative on Sharing All Influenza Data (GISAID) (Shu and McCauley, 2017). These high-quality genomic sequences of SARS-CoV-2 were screened out under the following criteria: 1) the genome is full-length; 2) exclusion of the sequences with unsolved nucleotides (i.e continuous Ns). Meanwhile, we downloaded the high-quality genomic sequences for “Variants of concern” that accord with above conditions: Alpha (B.1.1.7)with 108791 sequences; Beta (B.1.351) with 13871 sequences and Gamma (P.1) with 18808 sequences from May 1 to June 15, 2021; Delta (B.1.617.2) with 302879 sequences as of August 11, 2021; Omicron with 20356 sequences from November to December 22, 2021. “Variants of interest” include Lambda (C.37) with 627 sequences and Mu (B.1.621) with 2771 sequences until September 11, 2021 (**Figure S1**).

### Sequence Processing

To do unbiased genomic variation analysis, we further filtered and deleted those sequences with more than 50 consecutive N bases (50NNNs or 50nnns). Finally, 145,044 SARS-CoV-2 sequences were screened out. There were 92214, 6341, 13655, 211740, 2333, 345 and 2276 sequences selected for Alpha (B.1.1.7), Beta (B.1.351), Gamma (P.1), Delta (B.1.617.2), Omicron (B.1.1.529), Lambda (C.37) and Mu (B.1.621), respectively.

The SARS-CoV-2 genome of NC_045512.2 (Wu et al., 2020) was utilized as the reference sequence. Multiple sequences alignments were performed using the progressive method (FFT-NS-2) implemented in MAFFT (version 7.4) (Katoh et al., 2002). The whole genome mutation analysis was carried out used the pipeline provided by CoVa (version 0.2) software (Ali et al., 2020). For substitution analysis, we first deleted the gap generated in the multi-sequence alignments based on the ref NC_045512.2 and then used the CoVa pipeline for substitution calculation (**Figure S1**).

We summarize the mutation frequency of each mutant sequences. In order to avoid false positive results caused by errors in sequencing and multi-sequence alignments, we only analyzed the mutations with frequency greater than or equal to 10.

### Plasmid Constructs

Standard plasmids were purchased from Sangon Biotech (Shanghai). Point mutations were introduced into standard plasmids by PCR-mediated mutagenesis, with appropriate primers (**Table S1**) containing the desired nucleotide changes and subsequently selected by DpnI digestin.

### qPCR Analysis

The plasmids was amplified by a fast two-step amplification program using Taq Pro HS Universal Probe Master Mix (Vazyme Biotech Co., Ltd.).

## Results

### Mutations of SARS-CoV-2 Lead to Decrease in Detection Efficiency of qPCR

To explore the impact of mutations on qPCR detection of the circulating strains of SARS-CoV-2, we downloaded and analyzed the overall mutations and deletions of 89,791 high-quality SARS-CoV-2 sequences from the GISAID database (detailed information in methods).

**Figure 1:**
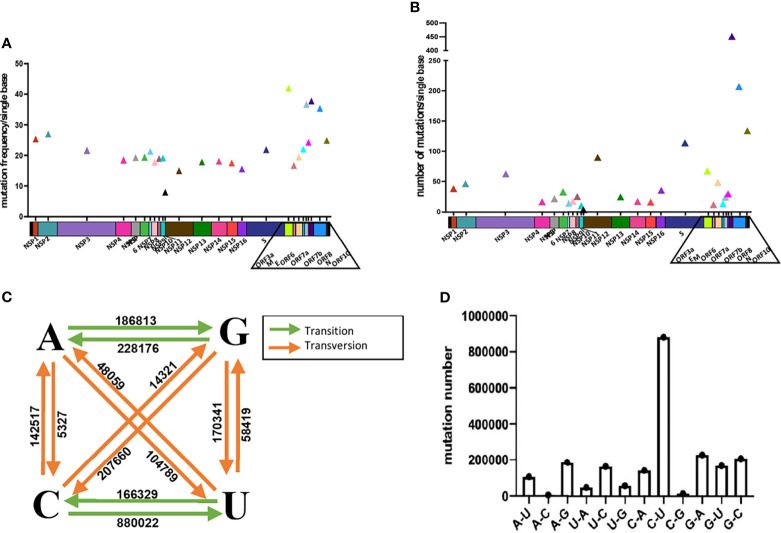
Analysis of the mutations in SARS-CoV-2. Analyze the average mutation frequency **(A)** and number **(B)** of mutations per base of 89,791 SARS-CoV-2 sequences in 26 viral genes. Average mutation frequency = The total number of mutation sites of gene in 89,791 SARS-CoV-2 sequences/the total number bases of the gene. Average mutation number = The total number of mutations of gene in 89,791 SARS-CoV-2 sequences/the total number bases of the gene. **(C, D)** Analysis of base selection for mutations in 89,791 SARS-CoV-2 sequences.

We first made an analysis of the overall mutations. We calculated the mutation frequency and quantity at each site along the SARS-CoV-2 genomes in comparison with the wildtype strain (**Figures 1A, B**). In general, the mutation frequency and quantity of the genomic regions encoding structural proteins and accessory proteins are higher than that of non-structural proteins. In the regions of ORF3a, ORF7a, ORF8 and N genes, mutations were observed at the positions of more than 30% of the bases, indicating a high propencity of mutations throughout the genes. For example, we found that there are mutations at 445 sites in N gene, which account for 35.3% of the total 1260 nucleotides. In the analyzed sequences, the average number of mutations per base of S, ORF8, N and ORF10 genes exceeds 100, indicating that, on average, at least 100 of the analyzed sequences contain a mutation at one nucleotide position of the genes. In the S gene, we found a total of 434360 mutations in the 89,791 SARS-CoV-2 sequences, with an average of 113.6 sequences having mutation at one site of the S gene (**Figures 1A, B**). S and N genes belong to the main targets for qPCR detections. These two genes have a high probability of mutation, which may have a potential impact on qPCR detection.

Next, we analyzed the preference of the base mutations and found that the ratio of transition to transversion mutation is about 2:1. And the results show that the most frequently mutated base is cytosine (C) followed by guanine (G), uracil (U) and adenine (A). C and G mutations accounted for 46.85% and 27.39% of the total number of mutations (**Figures 1C, D**). The mutation from C to U is most frequent and accounted for 39.77% of the total number of mutations. A→C and C→G are the two least mutations, accounting for only 0.24% and 0.64% of the total number of mutations.

Then we compared mutations with the primer/probe sequences of qPCR most commonly used in the commercial providers or public institutions (13 pairs from China, and 15 pairs from other countries were selected) (**Figure S2** and **Tables S2**, **S3**). The positions and numbers of nucleotide substitutions in more than 25 sequences and all deletions in the regions corresponding to the primer/probe sequences in **Table S2** and **Table S3**. We found that all the primer/probe-targeting sequences have varying degrees of mutations.

To explore the impact of these mutations on detection efficiency, we divide the mutations on the targets of primers/probes into 3 types:the mutation on the probe, the last two base mutation at the 3’-end of the primer and the mutation at other positions of primer.

**Figure 2:**
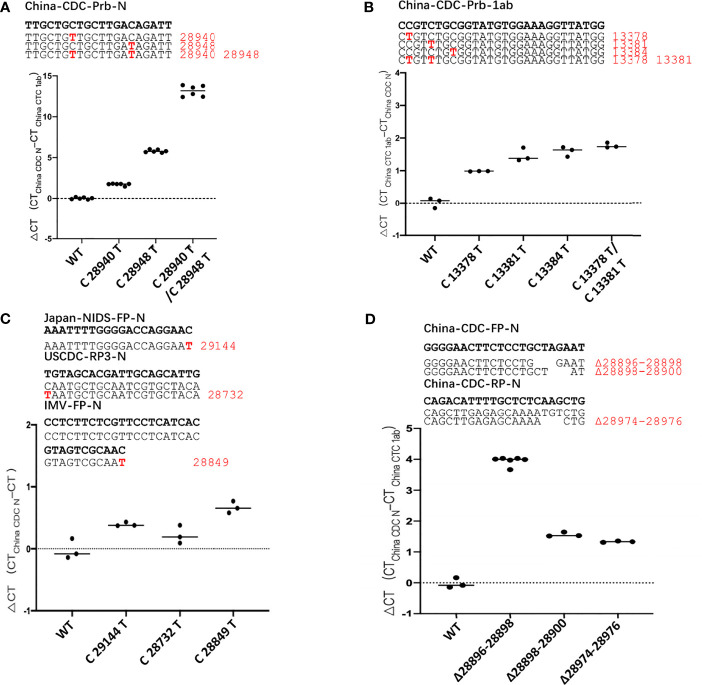
The mutation of SARS-CoV-2 leads to poor detection of qPCR. ΔCt values for testing of the standard plasmid and the mutant plasmid **(A, B)** mutations in the probe, **(C)** mutations in the 3’-end of primer, **(D)** deletion mutant on primer using primer/probe sets by qPCR. The red letters represent the mutation bases, and the red numbers represent the mutation positions. All plasmid used in the figure are 8.5 x 10^2^ copies.

We took the two pairs of primer/probe sets of China CDC, targeting the 1ab and the N gene as an example, and generated sequence template with single mutations and multi-site mutations that exist in the primer/probe target sequences on standard plasmids. These mutants are listed in **Table S2**. We detected the difference of detection efficiency between the wildtype (wt) and the mutant sequences by qPCR. The results showed that the detection sensitivity of the mutations on the probe target was 3-32 times less than that of the wildtype sequence (**Figures 2A, B**). All mutations on the probe target would affect the detection efficiency of qPCR, and the detection sensitivity of the double-mutation template was 9900 times less than that of the wt sequence. Most individual mutations in the primer targets basically did not affect the detection efficiency of qPCR, except for some cases of multiple mutations (**Figures S3**, **S4**). In particular, the multiple mutations at the 3’-end of the primer affected the detection efficiency, and the closer the mutation is to 3’-end of primer, the greater the impact (**Figure S4**).

Then, we found that there are mutations in the 3’-end of primers in the three primer/probe sets (Japan-NIDS, USCDC, Institute of Microbiology and Virology (IMV)) with target sites on the N gene and constructed the corresponding site mutations on N gene sequence (**Table S2**). We used qPCR to detect the difference of detection efficiency between the wt and the mutant sequences (**Figure 2C**). It was found that a single mutation on 3’-end of primer had little effect on detection. According to previous reports, the degree of mismatch of 3’-end of primer that affects the detection efficiency, especially the mismatches of A: A, G: A and C:C cause the detection efficiency to be markedly reduced (Kwok et al., 1990; Ghedira et al., 2009; Stadhouders et al., 2010; Rejali et al., 2018). But, in the transition of C→U, mismatches of T:G has the least influence on the efficiency of qPCR detection, compared to other types of mismatches (Rejali et al., 2018). Therefore, we substituted the last 3’-base of primer target with any of the other 3 bases and compared the detection efficiency of qPCR with primer/probe sets of Japan-NIDS FP-N, USCDC RP-N3, IMV-FP-N etc. (**Figure S5**). It was found that the mismatch of A:G, G:G, A:A, T:C, C:C and T:T had apparent influence on the efficiency of qPCR. However, the mismatch of T:G and C:A had little effect on the efficiency of qPCR. Coincidentally, we found that the 3’-end of primer mutation sites (Japan-NIDS FP-N, USCDC RP-N3, IMV-FP-N) are T:G and C:A mismatches. However, A:G, G:G, A:A, T:C, C:C and T:T mismatches at the 3’-end of primer may affect the detection efficiency of qPCR.

We also tested the impact of deletion mutants on detection efficiency, and we took the primer/probe sets of China CDC targeting the N gene as an example. We found that a number of variant sequences contain a 3’-base deletion on the forward primer target (28896-28898, 28898-28900), and a 3’-base deletion on the reverse primer target (28974-28976). The deletion mutants were generated and detected by the qPCR (**Figure 2D**). It was found that the detection sensitivity of the deletion mutants was 3-16 times less than that of the wt sequence. Finally, we analyzed the detection limit of wt and deletion mutants template sequences. We diluted the templates to the concentration of 100, 10, 1 and 0.1 copies/μl (**Figure S6**). The results showed that wt template could be detected at 1 copy/μl, but only 10 copies/μl for mutant sequence.

In general, through the above experiments, we have determined that mutations on the probe and 3’-end of primer sequences have a significant impact on qPCR detection, while mutations at the remaining positions have relatively little impact.

### The G28916T Mutation in the Delta Variant Severely Affected the Detection Efficiency and Detection Limit of the Primer/Probe Set of CP4 and CP5

The currently circulating SARS-CoV-2 variants include 5 VOCs (Alpha, Beta, Gamma, Delta and Omicron) and two VOIs (Lambda (C.37) and Mu (B.1.621)). We downloaded high-quality sequences of these SARS-CoV-2 variants from the GISAID database and compared mutations with the primer/probe sequences of 28 commonly used commercial qPCR kits (**Table S4**). We marked the mutation positions in the primer/probe targets with a mutation rate higher than 10% in **Table S4**.

The sequence corresponding to the primers/probes designed for the S gene had the largest number of mutations. Mutation C23604A was found in Alpha, Omicron and Mu variants, mutation C23604G was found in Delta variant, and mutation T23599G was found in Omicron variant, accounting for more than 99% of the total sequences. These mutations in the CP1 Prb-S target sequence may affect the detection efficiency of qPCR (**Table 1**). Among the 5 main epidemic strains, we also found that G22813T was in Beta and Omicron variant, A22812C was in Gamma variant, and C21762T was in Omicron variant, accounting for 92.6%, 99.6% and 98.5% respectively. All major mutations that may influence the qPCR detection are listed in **Table 1**.

**Table 1:** The point mutation of primer/probe sets in SARS-CoV-2 Variants.

**92,214 high quality SARS-CoV-2 Alpha variant**			
CP1 (sigma)	FP-S5	**CAGGTATATGCGCTAGTTATCAGAC**	23565-23589
	RP-S5	**CCAAGTGACATAGTGTAGGCAATG**	23638-23661
	Prb-S5	**AGACTAATTCTCCTCGGCGGGCACG** AGACTAATTCTC**A**TCGGCGGGCACG **(91682)** **Mutation rate：99.42%**	23592-23616
**6,341 high-quality SARS-CoV-2 Beta variants**			
CP3 (State Key Laboratory of Emerging Infectious Diseases)	FP-S	**CCTACTAAATTAAATGATCTCTGCTTTACT**	22712-22741
	RP-S	**CAAGCTATAACGCAGCCTGTA**	22849-22869
	Prb-S	**CGCTCCAGGGCAAACTGGAAAG** CGCTCCAGGGCAAACTGGAAA**T (5877)** **Mutation rate：92.68%**	22792-22813
**13,655 high-quality SARS-CoV-2 Gamma variants**			
CP3 (State Key Laboratory of Emerging Infectious Diseases)	FP-S	**CCTACTAAATTAAATGATCTCTGCTTTACT**	22712-22741
	RP-S	**CAAGCTATAACGCAGCCTGTA**	22849-22869
	Prb-S	**CGCTCCAGGGCAAACTGGAAAG** CGCTCCAGGGCAAACTGGAA**C**G **(13609)** **Mutation rate：99.66%**	22792-22813
**211,740 high-quality SARS-CoV-2 Delta variants**			
CP4 (Da An Gene of Sun Yat-sen University)	FP-N	**AAGAAATTCAACTCCAGGCAGC**	28855-28876
	RP-N	**GCTGGTTCAATCTGTCAAGCAG**	28940-28961
	Prb-N	**TCACCGCCATTGCCAGCCA** TGGCTGGCAATGGCGGTGA TGGCTGGCAATGGC**T**GTGA **(166809)** **Mutation rate：78.78%**	28902-28920
CP5 (Institute of Microbiology and Virology)	FP-N	**CCTCTTCTCGTTCCTCATCACGTAGTCGCAAC**	28818-28849
	RP-N	**AGTGACAGTTTGGCCTTGTTGTTGTTGGCCTT**	28983-29014
	Prb-N	**CCTGCTAGAATGGCTGGCAATGGCGGTGA** CCTGCTAGAATGGCTGGCAATGGC**T**GTGA **(166809)** **Mutation rate：78.78%**	28892-28920
CP1 (sigma)	FP-S5	**CAGGTATATGCGCTAGTTATCAGAC**	23565-23589
	RP-S5	**CCAAGTGACATAGTGTAGGCAATG**	23638-23661
	Prb-S5	**AGACTAATTCTCCTCGGCGGGCACG** AGACTAATTCTC**G**TCGGCGGGCACG **(211272)** **Mutation rate：99.78%**	23592-23616
**2,333 high-quality SARS-CoV-2 Omicron variants**			
Institute	Name	Sequence	Position
CP6(USCDC)	FP-N1	**GACCCCAAAATCAGCGAAAT**	28287−28306
	RP-N1	**TCTGGTTACTGCCAGTTGAATCTG** CATATTCAACTGGCAGTAACCAGA	28335−28358
	Prb-N1	**ACCCCGCATTACGTTTGGTGGACC** AC**T**CCGCATTACGTTTGGTGGACC **(2297)** **Mutation rate：98.45%**	28309−28332
CP2 (Northwell Health Laboratories)	FP-S	**TCAACTCAGGACTTGTTCTTAC**	21710-21731
	RP-S	**TGGTAGGACAGGGTTATCAAAC** GTTTGATAACCCTGTCCTACCA	21796-21817
	Prb-S	**TGGTCCCAGAGACATGTATAGCAT** ATGCTATACATGTCTCTGGGACCA ATG**T**TATACATGTCTCTGGGACCA **(2297)** **Mutation rate：98.45%**	21759-21782
CP1 (Sigma)	FP-S5	**CAGGTATATGCGCTAGTTATCAGAC**	23565-23589
	RP-S5	**CATTGCCTACACTATGTCACTTGG** CCAAGTGACATAGTGTAGGCAATG	23638-23661
	Prb-S5	**AGACTAATTCTCCTCGGCGGGCACG** AGACTAA**G**TCTCCTCGGCGGGCACG **(2315)** **Mutation rate：99.23%** AGACTAATTCTC**A**TCGGCGGGCACG **(2312)** **Mutation rate：99.10%**	23592-23616
**345 high-quality SARS-CoV-2 Lambda variants**			
CP4 (Da An Gene Co., Ltd. of Sun Yat-sen University)	FP-N	**AAGAAATTCAACTCCAGGCAGC**	28855-28876
	RP-N	**GCTGGTTCAATCTGTCAAGCAG** CTGCTTGACAGATTGAACCAGC	28940-28961
	Prb-N	**TCACCGCCATTGCCAGCCA** TGGCTGGCAATGGCGGTGA TGGCTGGCAAT**T**GCGGTGA **(333)** **Mutation rate：96.52%**	28902-28920
CP5 (Institute of Microbiology and Virology)	FP-N	**CCTCTTCTCGTTCCTCATCACGTAGTCGCAAC** CCTCTTCTCGTTCCTCATCACGTAGTCGCAA**T (91)** **Mutation rate：26.38%**	28818-28849
	RP-N	**AGTGACAGTTTGGCCTTGTTGTTGTTGGCCTT** AAGGCCAACAACAACAAGGCCAAACTGTCACT	28983-29014
	Prb-N	**CCTGCTAGAATGGCTGGCAATGGCGGTGA** TCTGCTAGAATGGCTGGCAATGGCGGTGA TCTGCTAGAATGGCTGGCAAT**T**GCGGTGA **(333)** **Mutation rate：96.52%**	28892-28920
**2,276 high-quality SARS-CoV-2 Mu variants**			
CP1 (sigma)	FP-S5	**CAGGTATATGCGCTAGTTATCAGAC**	23565-23589
	RP-S5	**CCAAGTGACATAGTGTAGGCAATG** CATTGCCTACACTATGTCACTTGG	23638-23661
	Prb-S5	**AGACTAATTCTCCTCGGCGGGCACG** CGACTAATTCTC**A**TCGGCGGGCACG **(2270)** **Mutation rate：99.74%**	23592-23616

The red letters represent the mutated bases. The red number represents the total number of mutation sequences.

**Figure 3:**
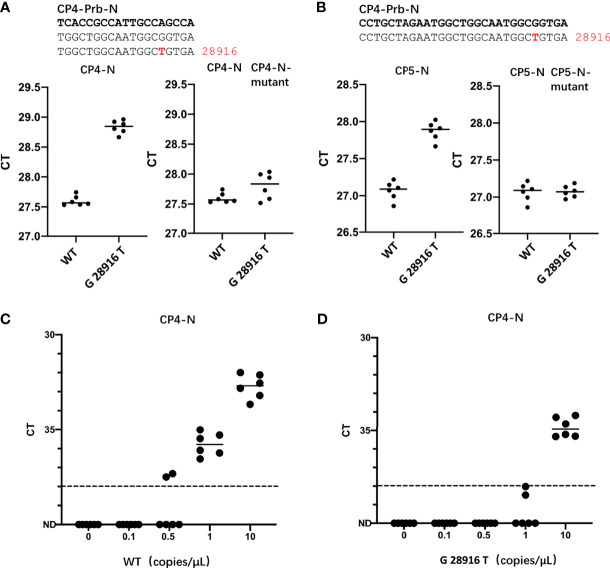
The G28916T mutation in the Delta variant severely affected the detection efficiency and detection limit of the primer/probe set of CP4 and CP5. **(A, B)** Ct values for testing of the standard plasmid and the G28916T mutant plasmid using CP3 and CP4 by qPCR. The plasmid used in Figures 3A, B are 8.5 x 10^2^ copies. **(C, D)** The lower detection limit of primer/probe, each primer-probe set uses 6 replicates, which contains standard and 28916 mutant plasmids, diluted according to 10, 1, 0.5 and 0.1 copies/ul. The red letters represent the mutated bases. The red number represents the position of the mutation.

In the currently most prevalent SARS-CoV-2 variant, the Delta variant, we found that there are 166809 sequences where the G at position 28916 was mutated to T, among the 211740 sequences in total, accounting for 78.78%. This mutation exists in the target sequence of CP4 and CP5 probes of the N gene (**Table 1**). Therefore, we generated the G28916T mutation on the N gene template. We used qPCR to detect the difference of detection efficiency between the wt and the mutant sequences. It was found that the detection sensitivity of the mutations on probe was 2.35 and 1.74 times less than that of the wt sequence (**Figures 3A, B**). Next, we changed the probe sequences of CP4 and CP5 to match with the mutant sequence. We found that if the G28916T was matched with primer sequence, the detection efficiency of qPCR was not affected (**Figures 3A, B**). Finally, we analyzed the detection limit of wt and mutant template sequences using the primer/probe CP4 for N gene. We diluted the templates to the concentration of 10, 1, 0.5 and 0.1 copies/μl (**Figures 3C, D**). The results showed that CP4 could detect the samples of 1 copies/μl for wt template, but only 10 copies/μl for mutant sequence. This showed that the mutation of G28916T in Delta variant increases the detection limit of the N gene primer/probe set of CP4 and CP5 by 10 times. Meanwhile, mutation G28913T and C28849T were found in Lambda variant, accounting for 96.52% and 26.38% respectively, which also affect the detection efficiency of CP4 and CP5 (**Table 1**).

## Discussion

In summary, we found that primers/probes targeting the S gene and N gene are commonly used for qPCR detection of SARS-CoV-2, and the detection efficiency of various SARS-CoV-2 variants may be significantly reduced if the original primer/probe sets are used. For efficient detection of the newly emerging SARS-CoV-2 variants, the mutations of the qPCR target sequence should be closely monitored, and the primers/probes sequence be correspondingly optimized in time to ensure the detection sensitivity and efficiency for various SARS-CoV-2 variants.

## Data Availability Statement

The original contributions presented in the study are included in the article/**Supplementary Material**. Further inquiries can be directed to the corresponding author.

## Author Contributions

DG and LC designed the research. LC, TX, YJ, and SH performed the experiments. LC, XL, HP, and CL analyzed the data. DG and LC wrote and revised the manuscript. All authors contributed to the article and approved the submitted version.

## Conflict of Interest

The authors declare that the research was conducted in the absence of any commercial or financial relationships that could be construed as a potential conflict of interest.

## Publisher’s Note

All claims expressed in this article are solely those of the authors and do not necessarily represent those of their affiliated organizations, or those of the publisher, the editors and the reviewers. Any product that may be evaluated in this article, or claim that may be made by its manufacturer, is not guaranteed or endorsed by the publisher.

## Supplementary Material

Supplementary Figure 1 Flow chart of sequence collection and screening.

Supplementary Figure 2 The primer/probe sequences of 28 commonly used commercial qPCR kits and their corresponding viral target genes. The 28 letters of A-AB correspond to 28 commonly used commercial qPCR kits, The position of the letter in SARS-CoV-2 genome corresponds to the target gene of the primer/probes.

Supplementary Figure 3 The mutation of forward/reverse on the primer pair basically has no effect on the efficiency of qPCR detection. ΔCt values for testing of the standard plasmid and the multiple mutations plasmid **(A)** mutations in China-CDC-FP-N, **(B)** China-CDC-RP-N, **(C)** China-CDC-1ab using China CDC 1ab and N primer/probe sets by qPCR. The red letters represent the mutation bases, and the red numbers represent the mutation positions. The red letters represent the mutated bases. The red number represents the position of the mutation. All plasmid used in the figure are 8.5 x 10^2^ copies.

Supplementary Figure 4 The multiple mutations close to the 3’-end of primer have a great impact on the efficiency of qPCR detection. ΔCt values for testing of the standard plasmid and the mutant plasmid on China CDC ORF1a primer using China CDC ORF1a and N primer/probe sets by qPCR. The red letters represent the mutation bases, and the red numbers represent the mutation positions. The red letters represent the mutated bases. The red number represents the position of the mutation. All plasmid used in the figure are 8.5 x 10^2^ copies.

Supplementary Figure 5 3’-end of primer mispairing influence detection efficiency of qPCR. We choose Japan-NIDS-FP-N **(A)**, USCDC-RP3-N **(B)**, IMV-FP-N **(C)**, USCDC-FP1-N **(D)**, USCDC-RP1-N **(E)**, China-CDC-FP-N **(F)**, USCDC-FP2-N **(G)**, China-CDC-RP-1ab **(H)** to construct four mutants (A, T, C, G) at 3’-end of primer, and detect the detection efficiency by qPCR on standard plasmid. The red letters represent the mutation bases. The red letters represent the mutated bases. All plasmid used in the figure are 8.5 x 10^2^ copies.

Supplementary Figure 6 The deletion mutation of SARS-CoV-2 affected the detection limit of the primer/probe set. **(A-D)** The lower detection limit of primer/probe, each primer-probe set uses 6 replicates, which contains standard and deletion mutant plasmids, diluted according to 100, 10, 1 and 0.1 copies/ul. The red letters represent the mutated bases. The red number represents the position of the mutation.
