# Supplementary material for: The Impact of Accumulated Mutations in SARS-CoV-2 Variants on the qPCR Detection Efficiency
Source: Front Cell Infect Microbiol. 2022 Jan 28;12:823306. doi: 10.3389/fcimb.2022.823306 (PMC8834649; doi:10.3389/fcimb.2022.823306)

Figure S1. Flow chart of sequence collection and screening

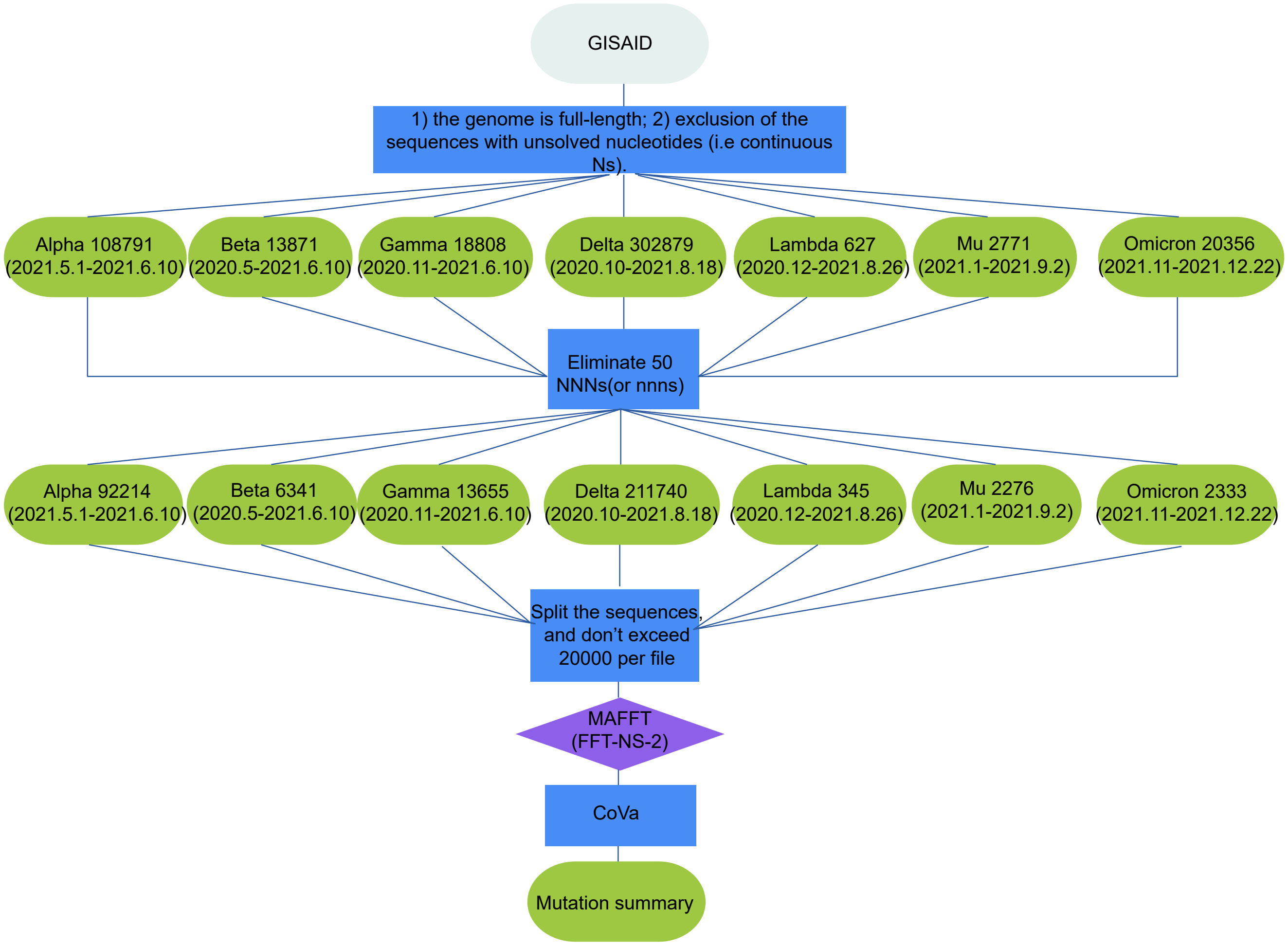

Figure S2. The primer/probe sequences of 28 commonly used commercial qPCR kits and their corresponding viral target genes

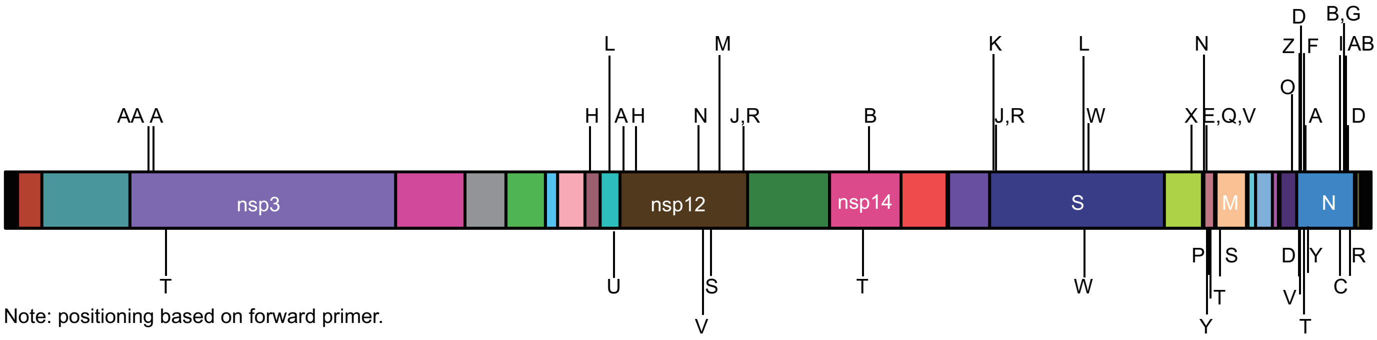

Note: positioning based on forward primer.

A: China CDC
B: HKU CDC
C: Japan NIDS
D: USCDC
E: Charité
F: Institute of Microbiology and Virology
G: The First Hospitalof Jilin University
H: Institut Pasteur
I: IPBCAMS
J: Boston Children’s Hospital
K: Northwell Health Laboratories
L: Anglia Ruskin Univeisity
M: Shanghai Public Health Clinical Center
N: University of Leipzig
O: University of Malaya
P: Department of Pulmonary and Critical Care Medicine
Q: Institute of Medical Microbiology, Virology and Hygiene (UKE)
R: State Key Laboratory of Emerging Infectious Diseases
S: Zheng Zhou Zhong Dao Biotechnology
T: Da An Gene of Sun Yat-sen University
U: Guangdong Pharmaceutical University
V: Sichuan Academy of Medical Sciences-Sichuan Provicial People's Hospital (SAMSPH)
W: Sigma
X: Hangzhou Qianji Biotechnology
Y: Xi'an Bioreal-coming BioMed Center
Z: Thailand NIH
AA: Shenzhen United Medical Technology
AB: Chaozhou Kaipu Biochemistry

Figure S3. The mutation of forward /reverse on the primer pair basically has no effect on the efficiency of qPCR detection

A

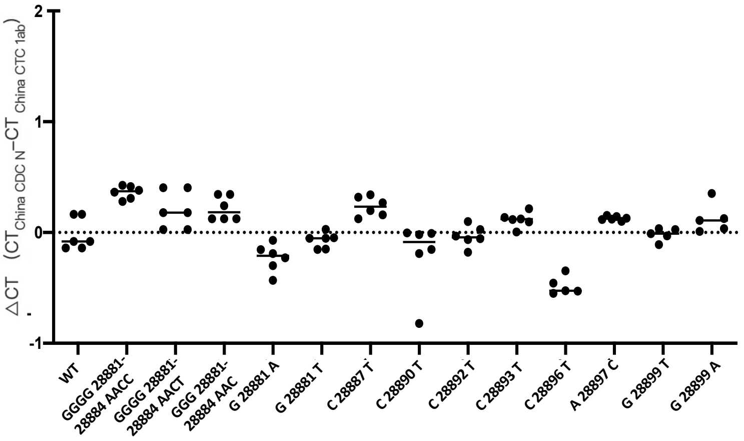

China-CDC-FP-N

GGGGAACTTCTCCTGCTAGAAT

AACGAACTTCTCCTGCTAGAAT 28881-28883
AACCAACTTCTCCTGCTAGAAT 28881-28884
AACTAACTTCTCCTGCTAGAAT 28881-28884
AGGGAACTTCTCCTGCTAGAAT 28881
TGGGAACTTCTCCTGCTAGAAT 28881
GGGGAA**T**TTCTCCTGCTAGAAT 28887
GGGGAACTT**T**TCCTGCTAGAAT 28890
GGGGAACTTCT**T**CTGCTAGAAT 28892
GGGGAACTTCTC**T**TGCTAGAAT 28893
GGGGAACTTCTCCTG**T**TAGAAT 28896
GGGGAACTTCTCCTGC**C**AGAAT 28897
GGGGAACTTCTCCTGCCA**T**AAT 28899
GGGGAACTTCTCCTGCCA**A**AAT 28899

B

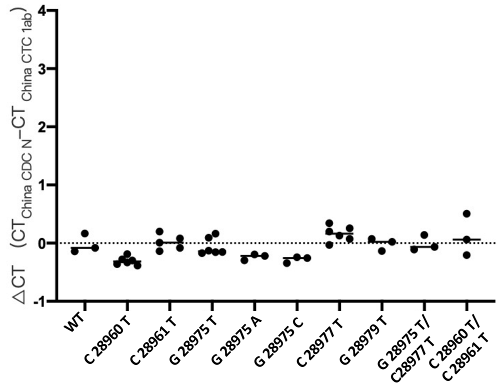

China-CDC-RP-N

CAGACATTTTGCTCTCAAGCTG

CAGCTTGAGAGCAAAATGTCTG
CA**T**CTTGAGAGCAAAATGTCTG28960
CAG**T**TTGAGAGCAAAATGTCTG28961
CAGCTTGAGAGCAAAAT**C**TCTG28975
CAGCTTGAGAGCAAAAT**T**TCTG28975
CAGCTTGAGAGCAAAAT**A**TCTG28975
CAGCTTGAGAGCAAAATAT**T**TG28977
CAGCTTGAGAGCAAAATATCT**T**28979

C

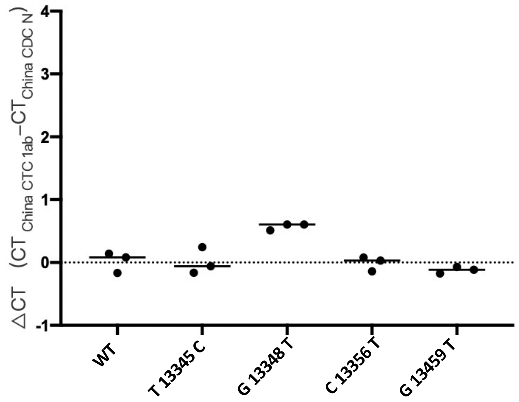

China-CDC-FP-1ab

CCCTGTGGGTTTTACACTTAA

CCC**C**GTGGGTTTTACACTTAA13345
CCCTGT**T**GGTTTTACACTTAA13348
CCCTGTGGGTTTTA**T**ACTTAA13356

China-CDC-RP-1ab

ACGATTGTGCATCAGCTGA

TCAGCTGATGCACAATCGT
TCAGCTGATGCACAATC**T**T13459

Figure S4. The multiple mutations close to the 3'end of primer have a great impact on the efficiency of qPCR detection

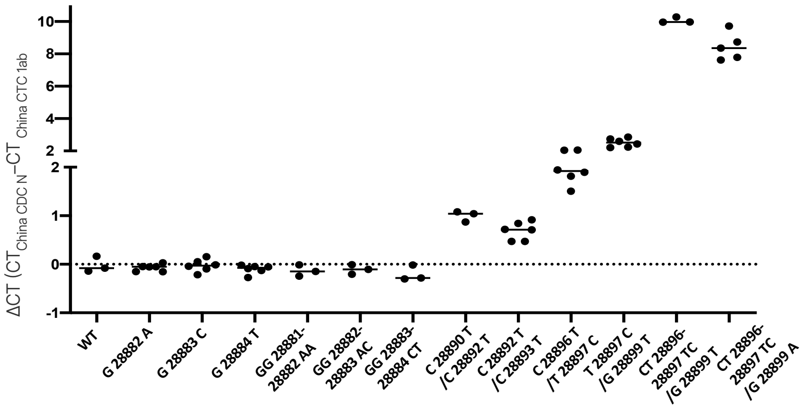

China-CDC-FP-N

```
GGGGAACTTCTCCTGCTAGAAT
AACGAACTTCTCCTGCTAGAAT   28882
AACCAACTTCTCCTGCTAGAAT   28883
AACTAACTTCTCCTGCTAGAAT   28884
AAGGAACTTCTCCTGCTAGAAT   28881-28882
GACGAACTTCTCCTGCTAGAAT   28882-28883
GGCTAACTTCTCCTGCTAGAAT   28883-28884
GGGGAACTTTTTCTGCTAGAAT   28890-28891
GGGGAACTTCTTTTGCTAGAAT   28892-28893
GGGGAACTTCTCCTGTCAGAAT   28896-28897
GGGGAACTTCTCCTGCCATAAT   28897 28899
GGGGAACTTCTCCTGTCATAAT   28896 28897 28899
GGGGAACTTCTCCTGTCAAAAT   28896 28897 28898
```

Figure S5. 3'end of primer mispairing influence detection efficiency of qPCR

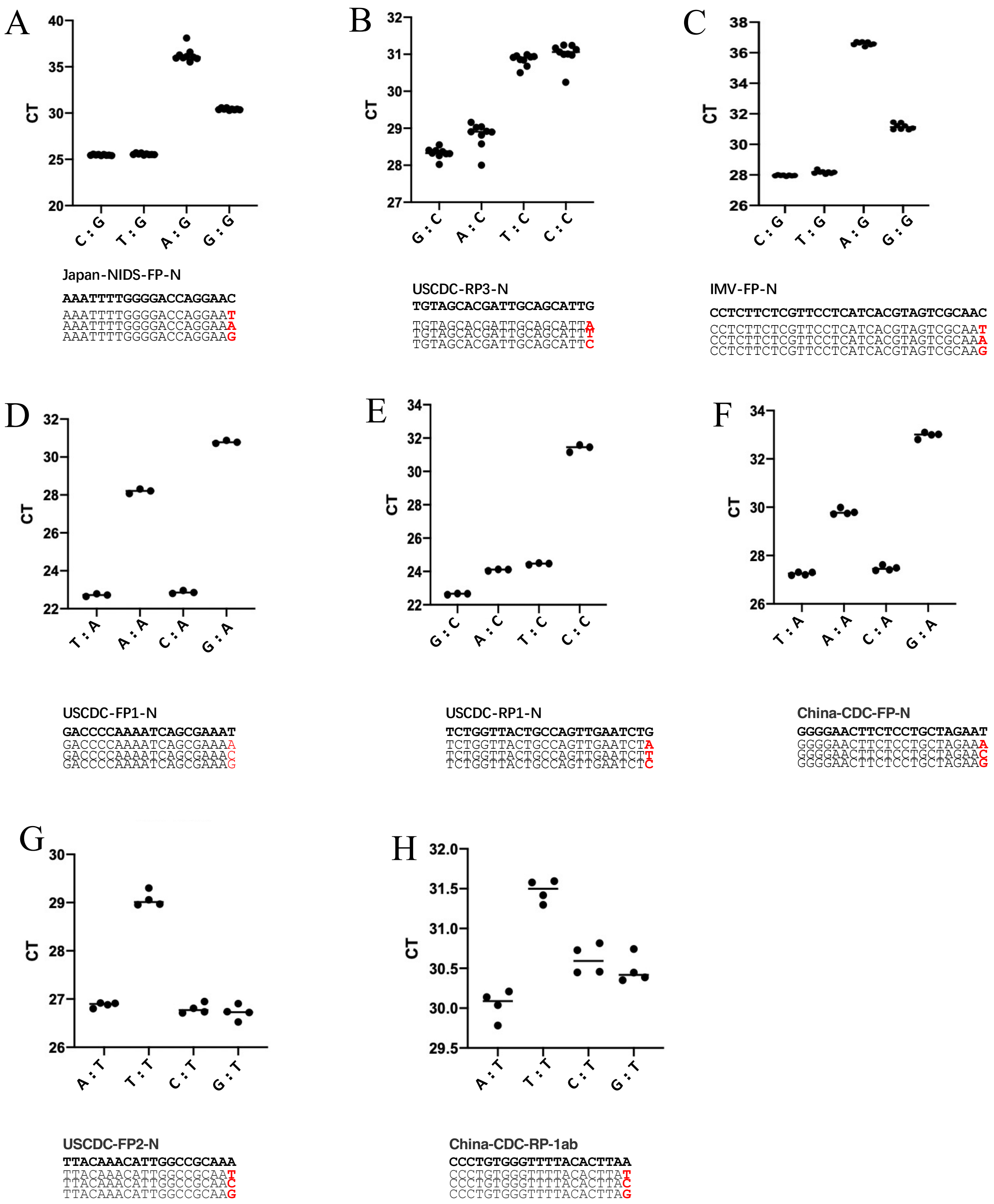

Figure S6. 3'end of primer mispairing influence detection efficiency of qPCR

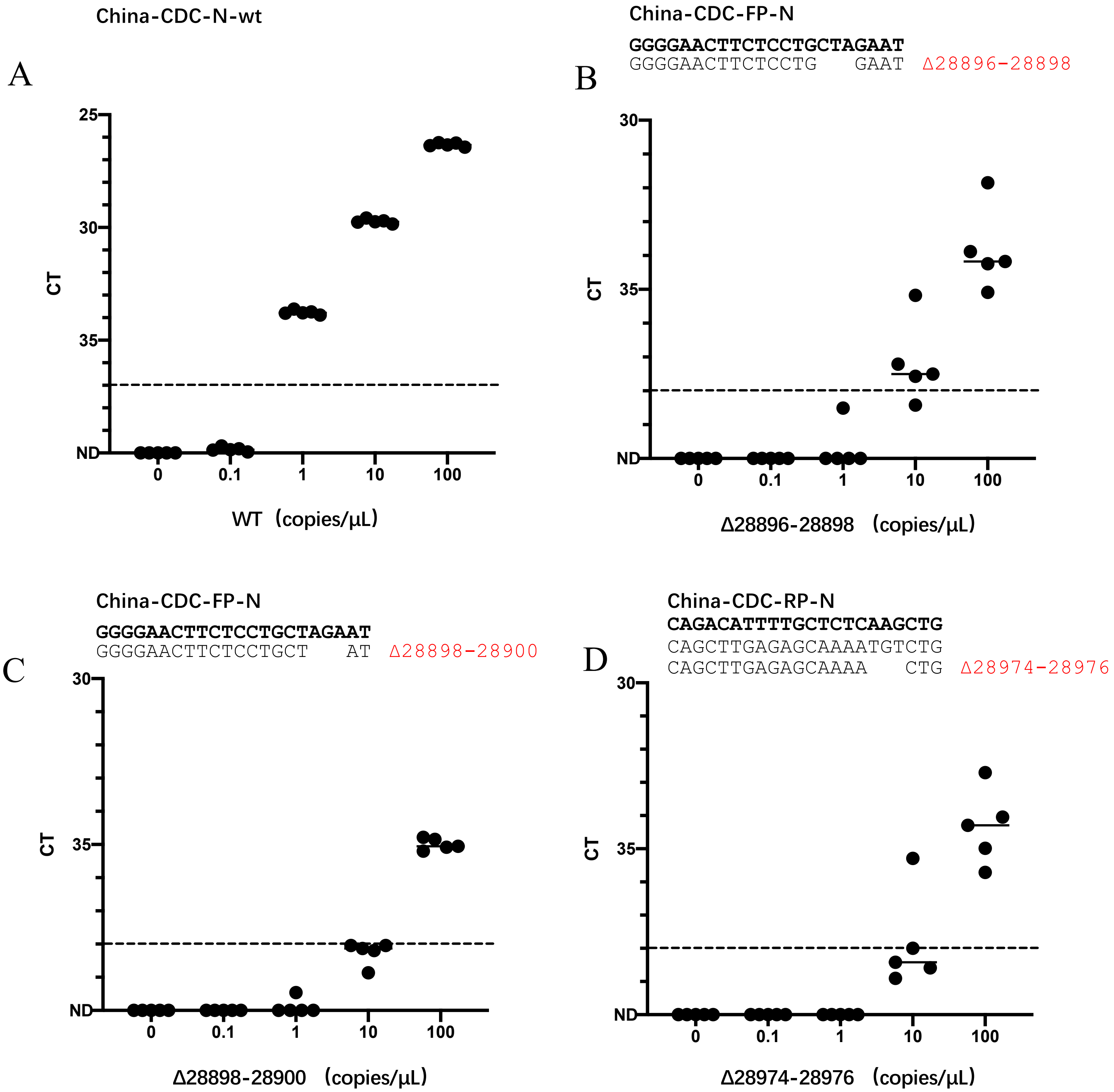

Supplement: Supplementary Figure 1 — Flow chart of sequence collection and screening. [file Image_1.pdf]
