# Supplementary material for: The Impact of Accumulated Mutations in SARS-CoV-2 Variants on the qPCR Detection Efficiency
Source: Front Cell Infect Microbiol. 2022 Jan 28;12:823306. doi: 10.3389/fcimb.2022.823306 (PMC8834649; doi:10.3389/fcimb.2022.823306)
Supplement: Supplementary file 2 [file Table_1.pdf]

Table S1. primers of mutations

MUTATION SITE	F	R
28881A	CAGGCAGCAGTA**A**GGGAACT	GGAGAAGTTCCC**T**TACTGCT
28882	AGGCAGCAGTAG**A**GGAACTT	AGGAGAAGTTCC**T**CTACTGC
28883	GGCAGCAGTAGG**C**GAACTTC	CAGGAGAAGTTC**G**CCTACTG
28884	GCAGCAGTAGGG**T**AACTTCT	GCAGGAGAAGTT**A**CCCTACT
28887	GCAGTAGGGGAA**T**TTCTCCT	CTAGCAGGAGAA**A**TTCCCCT
28890	GTAGGGGAACTT**T**TCCTGCT	ATTCTAGCAGGA**A**AAGTTCC
28892	AGGGGAACTTCT**T**CTGCTAG	CCATTCTAGCAG**A**AGAAGTT
28893	GGGGAACTTCTC**A**TGCTAGA	GCCATTCTAGCA**A**GAGAAGT
28896	GAACTTCTCCTG**T**TAGAATG	CCAGCCATTCTA**A**CAGGAGA
28897	AACTTCTCCTGC**C**AGAATGG	GCCAGCCATTCT**G**GCAGGAG
28899T	CCTGCTAGAATG**T**CTGGCAA	AGCCATTATAGC**A**GGAGAAG
28899A	CCTGCTAGAATG**A**CTGGCAA	AGCCATTATAGC**T**GGAGAAG
28940	CTTGCTTTGCTG**T**TGCTTGA	ATCTGTCAAGCA**A**CAGCAAA
28948	GCTGCTGCTTGA**T**AGATTGA	CTGGTTCAATCT**A**TCAAGCA
28940/<br>28948	1:CTTGCTTTGCTG**T**TGCTTGA<br>2: GCTGTTGCTTGA**T**AGATTGA	1: ATCTGTCAAGCA**A**CAGCAAA<br>2: CTGGTTCAATCT**A**TCAAGCA
28977	TGAGAGCAAAAT**T**TCTGGTA	GCCTTTACCAGA**A**ATTTTGC
28975	AGAGCAAAATGT**T**TGGTAAA	TGGCCTTTACCA**A**ACATTTT
28881-<br>28882	AGGCAGCAGTA**AA**GGAACTT	GGAGAAGTTCC**TT**TACTGCT
28882-<br>28883	GGCAGCAGTAG**AC**GAACTTC	AGGAGAAGTTC**GT**CTACTGC
28883-<br>28884	GCAGCAGTAGG**CT**AACTTCT	CAGGAGAAGTT**AG**CCTACTG
28881-<br>28884	GGCAGCAGTA**AACT**AACTTC	AGGAGAAGTTA**GTT**TACTGC
28890-<br>28891	TAGGGGAACTT**TG**CCTGCTA	ATTCTAGCAGG**CA**AAGTTCC
28892-<br>28893	GGGGAACTTCT**TT**TGCTAGA	CCATTCTAGCA**AA**AGAAGTT
28896-<br>28897	AACTTCTCCTG**TC**AGAATGG	CCAGCCATTCT**GA**CAGGAGA
28897<br>28899	ACTTCTCCTGC**C**A**T**AATGGC	TGCCAGCCATT**A**T**G**GCAGGA
28896<br>28897<br>28899	ACTTCTCCTG**TC**A**T**AATGGC	GCCAGCCATT**A**TG**A**CAGGAG
28896<br>28897<br>28899	ACTTCTCCTG**TC**AAAATGGC	GCCAGCCATT**A**TG**T**CAGGAG

28975<br>28977	GAGAGCAAAAT**T**T**T**TGGTAA	GGCCTTTACCA**A**A**A**ATTTTG
13348	TAATGACCCTGT**T**GGTTTTA	AAGTGTAAAACC**A**ACAGGGT
13378<br>13381	CAGTCTGTAC**T**GT**T**TGCGGT	ACATACCGCA**A**AC**A**GTACAG
13459	TGATGCACAATC**T**TTTTTAA	CCCGTTTAAAAA**A**GATTGTG
28881-<br>28884(2)	GGCAGCAGTA**AACC**AACTTC	AGGAGAAGTT**GGTT**TACTGC
28881T	CAGGCAGCAGTA**T**GGGAACT	GGAGAAGTTCCC**A**TACTGCT
28960	CAGATTGAACCA**T**CTTGAGA	TTTGCTCTCAAG**A**TGGTTCA
28961	AGATTGAACCAG**T**TTGAGAG	TTTTGCTCTCAA**A**CTGGTTC
28975 C	AGAGCAAAATGT**C**TGGTAAA	TGGCCTTTACCA**G**ACATTTT
28975 A	AGAGCAAAATGT**A**TGGTAAA	TGGCCTTTACCA**T**ACATTTT
28979	AGCAAAATGTCT**T**GTAAAGG	TGTTGGCCTTTA**A**CAGACAT
13345	TGCTAATGACCC**C**GTGGGTT	TGTAAAACCCAC**G**GGGTCAT
13384	CTGTACCGTCTG**T**GGTATGT	TTTCCACATACC**A**CAGACGG
28732	CAATCCTGCTAA**T**AATGCTG	GATTGCAGCATT**A**TTAGCAG
28849T	ACGTAGTCGCAA**T**AGTTCAA	ATTTCTTGAACT**A**TTGCGAC
28849A	ACGTAGTCGCAA**A**AGTTCAA	ATTTCTTGAACT**T**TTGCGAC
28849G	ACGTAGTCGCAA**G**AGTTCAA	ATTTCTTGAACT**C**TTGCGAC
29144T	GGGGACCAGGAA**T**TAATCAG	CTTGTCTGATTA**A**TTCCTGG
29144A	GGGGACCAGGAA**A**TAATCAG	CTTGTCTGATTA**T**TTCCTGG
29144G	GGGGACCAGGAA**G**TAATCAG	CTTGTCTGATTA**C**TTCCTGG
28732A	CAATCCTGCTAAAAATGCTG	GATTGCAGCATTTTTAGCAG
28732T	CAATCCTGCTAATAATGCTG	GATTGCAGCATTATTAGCAG
28732C	CAATCCTGCTAACAATGCTG	GATTGCAGCATTGTTAGCAG
28881-<br>28883	AGGCAGCAGTA**AAC**GAACTT	AGGAGAAGTTC**GTT**TACTGC
28960-<br>28961	AGATTGAACCA**TT**TTGAGAG	TTTGCTCTCAA**AA**TGGTTCA
13378	CACAGTCTGTAC**T**GTCTGCG	CATACCGCAGAC**A**GTACAGA
13381	AGTCTGTACCGT**T**TGCGGTA	CCACATACCGCA**A**ACGGTAC
28306A	AAATCAGCGAAAAGCACCCC	AATGCGGGGTGCTTTTCGCT
28306C	AAATCAGCGAAACGCACCCC	AATGCGGGGTGCGTTTCGCT
28306G	AAATCAGCGAAAGGCACCCC	AATGCGGGGTGCCTTTCGCT
28335A	CGTTTGGTGGACTCTCAGAT	GTTGAATCTGAGAGTCCACC
28335T	CGTTTGGTGGACACTCAGAT	GTTGAATCTGAGTGTCCACC
28335C	CGTTTGGTGGACGCTCAGAT	GTTGAATCTGAGCGTCCACC
28902A	CTCCTGCTAGAAAGGCTGGC	CCATTGCCAGCCTTTCTAGC
28902C	CTCCTGCTAGAACGGCTGGC	CCATTGCCAGCCGTTCTAGC
28902G	CTCCTGCTAGAAGGGCTGGC	CCATTGCCAGCCCTTCTAGC
29182T	ACATTGGCCGCATATTGCAC	AAATTGTGCAATATGCGGCC
29182C	ACATTGGCCGCACATTGCAC	AAATTGTGCAATGTGCGGCC

29182G	ACATTGGCCGCAGATTGCAC	AAATTGTGCAATCTGCGGCC
13362T	GTTTTACACTTATAAACACA	CAGACTGTGTTTATAAGTGT
13362C	GTTTTACACTTACAAACACA	CAGACTGTGTTTGTAAGTGT
13362G	GTTTTACACTTAGAAACACA	CAGACTGTGTTTCTAAGTGT
13356	AGCTGATGCACATTCGTTTT	GTTTAAAAACGAATGTGCAT
28916	GCTGGCAATGGCTGTGATGC	GAGCAGCATCACAGCCATTG
Δ28896-28898	CTTCTCCTGGAATGGCTGGCAATGGCG	CAGCCATTCCAGGAGAAGTTCCCCTAC
Δ28898-28900	TCTCCTGCTATGGCTGGCAATGGCGGT	GCCAGCCATAGCAGGAGAAGTTCCCCT
Δ28974-28976	AGAGCAAAACTGGTAAAGGCCAACAAC	CTTTACCAGTTTTGCTCTCAAGCTGGT
