# Supplementary material for: The Impact of Accumulated Mutations in SARS-CoV-2 Variants on the qPCR Detection Efficiency
Source: Front Cell Infect Microbiol. 2022 Jan 28;12:823306. doi: 10.3389/fcimb.2022.823306 (PMC8834649; doi:10.3389/fcimb.2022.823306)
Supplement: Supplementary file 3 [file Table_2.pdf]

Table S2. The point mutation of primer/probe sets in the sequence of SARS-CoV-2 (89,791 high-quality SARS-CoV-2 sequences)

Institute	Name	Sequence	Position
China CDC	FP-N	**GGGGAACTTCTCCTGCTAGAAT**<br>AACGAACTTCTCCTGCTAGAAT (47300)<br>AACCAACTTCTCCTGCTAGAAT (320)<br>AACTAACTTCTCCTGCTAGAAT (85)<br>AGGGAACTTCTCCTGCTAGAAT (79)<br>TGGGAACTTCTCCTGCTAGAAT (28)<br>GGGGAATTTCTCCTGCTAGAAT (1803)<br>GGGGAACTTTTCCTGCTAGAAT (172)<br>GGGGAACTTCTTCTGCTAGAAT (169)<br>GGGGAACTTCTCTTGCTAGAAT (208)<br>GGGGAACTTCTCCTGTTAGAAT (108)<br>GGGGAACTTCTCCTGCCAGAAT (35)<br>GGGGAACTTCTCCTGCCATAAT (91)<br>GGGGAACTTCTCCTGCCAAAAT (25)	28881–28902
	RP-N	**CAGACATTTTGCTCTCAAGCTG**<br>CAGCTTGAGAGCAAAATGTCTG<br>CATCTTGAGAGCAAAATGTCTG (42)<br>CAGTTTGAGAGCAAAATGTCTG (60)<br>CAGCTTGAGAGCAAAATCTCTG (3035)<br>CAGCTTGAGAGCAAAATTTCTG (739)<br>CAGCTTGAGAGCAAAATATCTG (245)<br>CAGCTTGAGAGCAAAATATTTG (44269)<br>CAGCTTGAGAGCAAAATATCTT (26)	28958–28979
	Prb-N	**TTGCTGCTGCTTGACAGATT**<br>TTGCTGTTGCTTGACAGATT (32)<br>TTGCTGCTGCTTGATAGATT (202)	28934–28953
	FP-ORF1ab1	**CCCTGTGGGTTTTACACTTAA**<br>CCCCGTGGGTTTTACACTTAA (40)<br>CCCTGTTGGTTTTACACTTAA (47)<br>CCCTGTGGGTTTTATACTTAA (43)	13342–13362
	RP-ORF1ab1	**ACGATTGTGCATCAGCTGA**<br>TCAGCTGATGCACAATCGT<br>TCAGCTGATGCACAATCTT (53)	13442–13460
	Prb-ORF1ab1	**CCGTCTGCGGTATGTGGAAAGGTTATGG**<br>CTGTCTGCGGTATGTGGAAAGGTTATGG (64)<br>CCGTTTGCGGTATGTGGAAAGGTTATGG (26)<br>CCGTCTGTGGTATGTGGAAAGGTTATGG (250)	13377–13404
	FP-ORF1ab2	**AGAAGATTGGTTAGATGATGATAGT**<br>AGAAGACTGGTTAGATGATGATAGT (32)	3193-3217
	RP-	**TTCCATCTCTAATTGAGGTTGAACC**	3286-3310

	ORF1ab2	GGTTCAACCTCAATTAGAGATGGAA<br>GGTTCAACCTCAATTA**A**AGATGGAA **(94)**<br>GGTTCAACCTCAATTAGAGAT**T**GAA **(43)**	
	Prb-ORF1ab2	**TCCTCACTGCCGTCTTGTTGACCA**<br>TGGTCAACAAGACGGCAGTGAGGA<br>T**A**GTCAACAAGACGGCAGTGAGGA **(51)**<br>TG**T**TCAACAAGACGGCAGTGAGGA **(190)**<br>TGGTC**T**ACAAGACGGCAGTGAGGA **(37)**<br>TGGTCAACAA**T**ACGGCAGTGAGGA **(165)**<br>TGGTCAACAAGA**T**GGCAGTGAGGA **(53)**	3229-3252
HKU CDC	FP-N	**TAATCAGACAAGGAACTGATTA**<br>TAA**C**CAGACAAGGAACTGATTA **(41)**<br>TAAT**T**AGACAAGGAACTGATTA **(289)**<br>TAATCAGACAAGGAA**T**TGATTA **(33)**<br>TAATCAGACAAGGAACT**T**ATTA **(66)**	29145–29166
	RP-N	**CGAAGGTGTGACTTCCATG**<br>CATGGAAGTCACACCTTCG<br>CATGGAAGTCA**T**ACCTTCG **(51)**<br>CATGGAAGTCACA**T**CTTCG **(34)**<br>CATGGAAGTCACAC**T**TTCG **(27)**<br>CATGGAAGTCACACC**C**TCG **(39)**<br>CATGGAAGTCACACCTT**T**G **(141)**<br>CATGGAAGTCACACCTTC**T** **(123)**<br>CATGGAAGTCACACCTTC**A** **(66)**	29235–29254
	Prb-N	**GCAAATTGTGCAATTTGCGG**<br>CCGCAAATTGCACAATTTGC<br>CC**T**CAAATTGCACAATTTGC **(384)**<br>CCGCAAATTGCACAATT**C**GC **(241)**<br>CCGCAAATTGCACAATTT**T**C **(32)**	29177–29196
HKU CDC	FP- nsp14	**TGGGGYTTTACRGGTAACCT**<br>TGGGGYTTTA**T**RGGTAACCT **(100)**<br>TGGGGYTTTACRGGTAAC**T**T **(71)**	18778–18797
	RP- nsp14	**AACRCGCTTAACAAAGCACTC**<br>GAGTGCTTTGTTAAGCGTGTT<br>GAGTGCTTTGTTAAG**T**GTGTT **(53)**	18889–18909
	Prb- nsp14	**TAGTTGTGATGCWATCATGACTAG**<br>TAGTTGTGAT**T**CWATCATGACTAG **(41)**	18849–18872
Japan NIDS	FP-N	**AAATTTTGGGGACCAGGAAC**<br>AAATTTTGGGGACCA**A**GAAC **(80)**<br>AAATTTTGGGGACCA**T**GAAC **(27)**<br>AAATTTTGGGGACCAGGAA**T** **(95)**	29125–29144
	RP-N	**TGGCAGCTGTGTAGGTCAAC**<br>GTTAACCTACACAGCTGCCA<br>GTT**A**ACCTACACAGCTGCCA **(548)**	29263–29282

		GTTGACCTA**T**ACAGCTGCCA (377)<br>GTTGACCTACA**T**AGCTGCCA (76)<br>GTTGACCTACACAGCTGC**T**A (78)	
	Prb-N	**ATGTCGCGCATTGGCATGGA**<br>ATGTC**T**CGCATTGGCATGGA (166)<br>ATGTCGC**A**CATTGGCATGGA (68)	29222–29241
USCDC	FP-N1	**GACCCCAAAATCAGCGAAAT**<br>GACCCCAAAATCAGCGAA**G**T (25)	28287–28306
	RP-N1	**TCTGGTTACTGCCAGTTGAATCTG**<br>CATATTCAACTGGCAGTAACCAGA<br>CA**T**ATTCAACTGGCAGTAACCAGA (63)<br>CAGATT**T**AACTGGCAGTAACCAGA (31)<br>CAGATTCAA**T**TGGCAGTAACCAGA (48)<br>CAGATTCAACT**T**GCAGTAACCAGA (46)	28335–28358
	Prb-N1	**ACCCCGCATTACGTTTGGTGGACC**<br>A**A**CCCGCATTACGTTTGGTGGACC (195)<br>A**T**CCCGCATTACGTTTGGTGGACC (111)<br>AC**T**CCGCATTACGTTTGGTGGACC (224)<br>ACC**T**CGCATTACGTTTGGTGGACC (100)<br>ACCC**T**GCATTACGTTTGGTGGACC (172)<br>ACCCC**G**CATTACGTTTGGTGGACC (61)<br>ACCCCGCATTA**T**GTTTGGTGGACC (43)<br>ACCCCGCATTAC**T**TTTGGTGGACC (290)<br>ACCCCGCATTAC**A**TTTGGTGGACC (180)<br>ACCCCGCATTACGTTTGGT**A**GACC (33)	28309–28332
	FP-N2	**TTACAAACATTGGCCGCAAA**<br>TTA**T**AAACATTGGCCGCAAA (183)<br>TTACAAA**T**ATTGGCCGCAAA (154)<br>TTACAAACATTGGCC**T**CAAA (384)	29164–29183
	RP-N2	**GCGCGACATTCCGAAGAA**<br>TTCTTTGGAATGTCGCGC<br>TTCTT**T**GGAATGTCGCGC (384)<br>TTCTTCGGAATGTC**T**CGC (384)<br>TTCTTCGGAATGTCGC**A**C (384)	29213–29230
	Prb-N2	**ACAATTTGCCCCCAGCGCTTCAG**<br>ACAATT**C**GCCCCCAGCGCTTCAG (241)<br>ACAATTT**T**CCCCCAGCGCTTCAG (32)<br>ACAATTTGC**T**CCCAGCGCTTCAG (255)<br>ACAATTTGCCCC**T**AGCGCTTCAG (217)<br>ACAATTTGCCCCCAGCGCTT**T**AG (146)	29188–29210
	FP-N3	**GGGAGCCTTGAATACACCAAAA**<br>GGGAGCCTT**T**AATACACCAAAA (167)<br>GGGAGCCTT**C**AATACACCAAAA (42)<br>GGGAGCCTTGAATA**T**ACCAAAA (98)	28681–28702

		GGGAGCCTTGAATACATCAAAA (75)	
	RP-N3	**TGTAGCACGATTGCAGCATTG**<br>TAATGCTGCAATCGTGCTACA<br>TAATGCTGCAATCGTGCTACA (27)<br>CAACGCTGCAATCGTGCTACA (354)<br>CAATGCTTCAATCGTGCTACA (53)<br>CAATGCTGCAATTGTGCTACA (153)<br>CAATGCTGCAATCGTTCTACA (59)	28732–287<br>52
	Prb-N3	**AYCACATTGGCACCCGCAATCCTG**<br>AYTACATTGGCACCCGCAATCCTG (263)<br>AYCATATTGGCACCCGCAATCCTG (78)<br>AYCACATTGGCATCCGCAATCCTG (31)<br>AYCACATTGGCACTCGCAATCCTG (30)<br>AYCACATTGGCACCCGTAATCCTG (69)<br>AYCACATTGGCACCCGCAACCCTG (30)<br>AYCACATTGGCACCCGCAATTCTG (122)<br>AYCACATTGGCACCCGCAATCTTG (376)<br>AYCACATTGGCACCCGCAATCCTT (29)	28704–287<br>27
	FP-N4	**CATGACGTTCGTGTT**<br>CATGATGTTCGTGTT (33)<br>CATGACGTTTGTGTT (61)<br>CATGACGTTCTTGTT (26)	28227-<br>28241
	RP-N4	**TCTGGTTACTGCCAGTTGAATCTG**<br>CAGATTCAACTGGCAGTAACCAGA<br>CATATTCAACTGGCAGTAACCAGA (63)<br>CAGATTTAACTGGCAGTAACCAGA (31)<br>CAGATTCAATTGGCAGTAACCAGA (48)<br>CAGATTCAACTTGCAGTAACCAGA (46)	28335-<br>28358
	Prb-N4	**ACCCCGCATTACGTTTGGTGG**<br>AACCCGCATTACGTTTGGTGG (195)<br>ATCCCGCATTACGTTTGGTGG (111)<br>ACTCCGCATTACGTTTGGTGG (224)<br>ACCTCGCATTACGTTTGGTGG (100)<br>ACCCTGCATTACGTTTGGTGG (172)<br>ACCCCTCATTACGTTTGGTGG (61)<br>ACCCCGCATTATGTTTGGTGG (43)<br>ACCCCGCATTACTTTTGGTGG (290)<br>ACCCCGCATTACATTTGGTGG (180)<br>ACCCCGCATTACGTTTGGTAG (33)	28309-<br>28329
Charité	FP-E	**ACAGGTACGTTAATAGTTAATAGCGT**<br>ATAGGTACGTTAATAGTTAATAGCGT (196)	26269-<br>26294
	RP-E	**ATATTGCAGCAGTACGCACACA**<br>TGTGTGCGTACTGCTGCAATAT	26360-<br>26381
	Prb-E	**ACACTAGCCATCCTTACTGCGCTTCG**	26332-

		ATACTAGCCATCCTTACTGCGCTTCG (29)	26357
Institute of Microbiology and Virology	FP-N	**CCTCTTCTCGTTCCTCATCACGTAGTCGCAAC**<br>CCTATTCTCGTTCCTCATCACGTAGTCGCAAC (90)<br>CCTCTTCTCGTTCCTTATCACGTAGTCGCAAC (141)<br>CCTCTTCTCGTTCCTCATCACGTATTCGCAAC (93)<br>CCTCTTCTCGTTCCTCATCACGTAGGCGCAAC (39)<br>CCTCTTCTCGTTCCTCATCACGTAGTCGTAAC (103)<br>CCTCTTCTCGTTCCTCATCACGTAGTCGCAAT (174)	28818-28849
	RP-N	**AGTGACAGTTTGGCCTTGTTGTTGTTGGCCTT**<br>AATGCCAACAACAACAAGGCCAAACTGTCACT (102)<br>AAGGCCAACAACAACAATGCCAAACTGTCACT (143)	28983-29014
	Prb-N	**CCTGCTAGAATGGCTGGCAATGGCGGTGA**<br>TCTGCTAGAATGGCTGGCAATGGCGGTGA (169)<br>CTTGCTAGAATGGCTGGCAATGGCGGTGA (208)<br>CCTGTTAGAATGGCTGGCAATGGCGGTGA (108)<br>CCTGCCAGAATGGCTGGCAATGGCGGTGA (35)<br>CCTGCTATAATGGCTGGCAATGGCGGTGA (91)<br>CCTGCTAAAATGGCTGGCAATGGCGGTGA (25)<br>CCTGCTAGAATTGCTGGCAATGGCGGTGA (117)<br>CCTGCTAGAATGTCTGGCAATGGCGGTGA (72)<br>CCTGCTAGAATGACTGGCAATGGCGGTGA (31)<br>CCTGCTAGAATGGTTGGCAATGGCGGTGA (103)<br>CCTGCTAGAATGGCTTGCAATGGCGGTGA (37)<br>CCTGCTAGAATGGCTGTCAATGGCGGTGA (64)<br>CCTGCTAGAATGGCTGGCAACGGCGGTGA (33)<br>CCTGCTAGAATGGCTGGCAATGGCGTTGA (28)	28892-28920
The First Hospitalof Jilin University	FP-N	**TAATCAGACAAGGAACTGATTA**<br>TAACCAGACAAGGAACTGATTA (41)<br>TAATTAGACAAGGAACTGATTA (289)<br>TAATCAGACAAGGAATTGATTA (33)<br>TAATCAGACAAGGAACTTATTA (66)	29145-29166
	RP-N	**CGAAGGTGTGACTTCCATG**<br>CATGGAAGTCACACCTTCG<br>CATGGAAGTCATACCTTCG (51)<br>CATGGAAGTCACATCTTCG (34)<br>CATGGAAGTCACACTTTCG (27)<br>CATGGAAGTCACACCCTCG (39)<br>CATGGAAGTCACACCTTTG (141)<br>CATGGAAGTCACACCTTCT (123)<br>CATGGAAGTCACACCTTCA (66)	29236-29254
	Prb-N	**GCAAATTGTGCAATTTGCGG**<br>CCGCAAATTGCACAATTTGC<br>CCTCAAATTGCACAATTTGC (384)<br>CCGCAAATTGCACAATTCGC (241)	29196-29177

		CCGCAAATTGCACAATTTTC (32)	
Institut Pasteur	FP-ORF1ab1	**ATGAGCTTAGTCCTGTTG**	12690-12707
	RP-ORF1ab1	**CTCCCTTTGTTGTGTTGT**<br>ACAACACAACAAAGGGAG<br>ATAACACAACAAAGGGAG (77)<br>ACAATACAACAAAGGGAG (69)<br>ACAACACAATAAAGGGAG(348)	12780-12797
	Prb-ORF1ab1	**AGATGTCTTGTGCTGCCGGTA**<br>AGATGTCTTGTGCTGCTGGTA(132)	12717-12737
	FP-ORF1ab2	**GGTAACTGGTATGATTTCG**<br>GGTTACTGGTATGATTTCG (79)<br>GGTAACTGGTATGATTTTG (36)	14080-14098
	RP-ORF1ab2	**CTGGTCAAGGTTAATATAGG**<br>CCTATATTAACCTTGACCAG<br>CCTATATTAACTTTGACCAG (89)<br>CCTATATTAACCTTGATCAG (61)<br>CCTATATTAACCTTGACTAG (86)	14167-14186
	Prb-ORF1ab2	**TCATACAAACCACGCCAGG**<br>TCATACAAACCAGGCCAGG (40)<br>TCATACAAACCATGCCAGG (29)<br>TCATACAAACCACTCCAGG (129)<br>TCATACAAACCACGCTAGG (1966)	14105-14123
IPBCAMS	FP-N	**AACACAAGCTTTCGGCAGAC**<br>AATACAAGCTTTCGGCAGAC (40)<br>AACACAAGCTTTTGGCAGAC (492)	29083-29102
	RP-N	**ACCTGTGTAGGTCAACCACG**<br>CGTGGTTGACCTACACAGGT<br>CTTGGTTGACCTACACAGGT (142)<br>CCTGGTTGACCTACACAGGT (54)<br>CATGGTTGACCTACACAGGT (37)<br>CGTGGTTAACCTACACAGGT (548)<br>CGTGGTTGACCTATACAGGT (377)<br>CGTGGTTGACCTACATAGGT (76)	29278-29259
	Prb-N	**CAGCGCTTCAGCGTTCTTCGGAATGTCGC**<br>TAGCGCTTCAGCGTTCTTCGGAATGTCGC (217)<br>CAGCGCTTCAGCGTTCTTTGGAATGTCGC (146)<br>CAGCGCTTCAGCGTTCTTCGGAATGTCTC (166)	29200-29228
Northwell Health Laboratories	FP-S	**TCAACTCAGGACTTGTTCTTAC**<br>TCAATTCAGGACTTGTTCTTAC (72)<br>TCAACTCAGGATTTGTTCTTAC (103)<br>TCAACTCAGGACTTTTTCTTAC (101)	21710-21731

		TCAACTCAGGACTTGTTT**T**TTAC **(335)**	
	RP-S	**TGGTAGGACAGGGTTATCAAAC**<br>GTTTGATAACCCTGTCCTACCA<br>GTTT**T**ATAACCCTGTCCTACCA **(95)**<br>GTTTG**C**TAACCCTGTCCTACCA **(249)**<br>GTTTG**G**TAACCCTGTCCTACCA **(67)**<br>GTTTGATAACCCTGT**A**CTACCA **(232)**<br>GTTTGATAACCCTGT**T**CTACCA **(138)**<br>GTTTGATAACCCTGTC**T**TACCA **(76)**<br>GTTTGATAACCCTGTCCTACC**G** **(59)**	21796-<br>21817
	Prb-S	**TGGTCCCAGAGACATGTATAGCAT**<br>ATGCTATACATGTCTCTGGGACCA<br>A**C**GCTATACATGTCTCTGGGACCA **(35)**<br>AT**T**CTATACATGTCTCTGGGACCA **(59)**<br>ATG**T**TATACATGTCTCTGGGACCA **(129)**<br>ATGC**C**ATACATGTCTCTGGGACCA **(79)**<br>ATGCTATA**T**ATGTCTCTGGGACCA **(37)**<br>ATGCTATACAT**A**TCTCTGGGACCA **(361)**<br>ATGCTATACATGT**T**TCTGGGACCA **(85)**<br>ATGCTATACATGTCT**T**TGGGACCA **(58)**<br>ATGCTATACATGTCTCT**A**GGACCA **(93)**	21759-<br>21782
Anglia Ruskin Univeisity	FP-nsp10	**GGATCAAGAATCCTTTGGTGG**<br>GGA**C**CAAGAATCCTTTGGTGG **(73)**	13213-<br>13233
	RP-nsp10	**GTCACAAAATCCTTTAGGATTTGGA**<br>TCCAAATCCTAAAGGATTTTGTGAC<br>**C**CCAAATCCTAAAGGATTTTGTGAC **(54)**<br>T**T**CAAATCCTAAAGGATTTTGTGAC **(31)**<br>TCCA**G**ATCCTAAAGGATTTTGTGAC **(34)**<br>TCCAAATCCTAA**G**GGATTTTGTGAC **(84)**<br>TCCAAATCCTAAAGGATTTTGTG**C**C **(36)**<br>TCCAAATCCTAAAGGATTTTGTGA**T** **(89)**	13273-<br>13297
	Prb-nsp10	**CATCGTGTTGTCTGTACTGCCGTTGCC**	13236-<br>13259
	FP-S	**CACCAGGAACAAATACTTC**	23358-<br>23376
	RP-S	**CCAAGTAGGAGTAAGTTGA**<br>TCAACTTACTCCTACTTGG<br>TCAACTTACT**T**CTACTTGG **(38)**	23443-<br>23461
	Prb-S	**CTTTATCAGGATGTTAACT**	23339-<br>23357
Shanghai Public Health	FP-ORF1ab	**TGATGATACTCTCTGACGATGCTGT**<br>TGATGATACT**T**TCTGACGATGCTGT **(53)**<br>TGATGATACTCTCTGA**T**GATGCTGT **(534)**	15704-<br>15728

Clinical Center	RP-ORF1ab	**CTCAGTCCAACATTTTGCTTCAGA**<br>TCTGAAGCAAAATGTTGGACTGAG	15823-15846
	Prb-ORF1ab	**ATGCATCTCAAGGTCTAGTG**<br>ATGC**G**TCTCAAGGTCTAGTG **(119)**<br>ATGCATCTCAAGGT**T**TAGTG **(87)**<br>ATGCATCTCAAGGTCTA**T**TG **(3255)**	15749-15768
University of Leipzig	FP-RdRp	**TATGCCATTAGTGCAAAGAATAGAGCTCGCAC**<br>TATGCCATTAGTGCAAAGAA**C**AGAGCTCGCAC **(3229)**	15076-15107
	RP- RdRp	**CAACCACCATAGAATTTGCTTGTTCCAATTAC**	15202-15233
	Prb- RdRp	**TCCTCTAGTGGCGGCTATTGATTTCAATAA**<br>TTATTGAAATCAATAGCCGCCACT**G**GAGGA **(34)**<br>TTATTGAAATCAATAGC**T**GCCACTAGAGGA **(35)**	15163-15192
	FP-E	**GAAGAGACAGGTACGTTAATAGTTAATAGCGTA**	26209-26241
	RP-E	**AAAAAGAAGGTTTTACAAGACTCACGTTAAC**<br>GTTAACGTGAGTCTTGTAAAACCTTCTTTTT<br>GTTAAC**T**TGAGTCTTGTAAAACCTTCTTTTT **(35)**	26383-26413
	Prb-E	**ATCGAAGCGCAGTAAGGATGGCTAG**<br>CTAGCCATCCTTACTGCGCTTCGAT	26335-26359
University of Malaya	FP-N	**TTGTTCGTTCTATGAAGACTTTTTAGAG**<br>TT**T**TTCGTTCTATGAAGACTTTTTAGAG **(173)**<br>TTGTTCGTTCTAT**T**AAGACTTTTTAGAG **(101)**<br>TTGTTCGTTCTATGA**G**GACTTTTTAGAG **(41)**<br>TTGTTCGTTCTATGAAGACT**C**TTTAGAG **(95)**	28196-28223
	RP- N	**TTTGATCGCGCCCCACTGCGTTCTCCATTC**<br>GAATGGAGAACGCAGTGGGGCGCGATCAAA<br>GAAT**A**GAGAACGCAGTGGGGCGCGATCAAA **(64)**<br>GAAT**C**GAGAACGCAGTGGGGCGCGATCAAA **(25)**<br>GAATGGAGAAC**A**CAGTGGGGCGCGATCAAA **(50)**<br>GAATGGAGAACGC**G**GTGGGGCGCGATCAAA **(193)**<br>GAATGGAGAACGCA**T**TGGGGCGCGATCAAA **(47)**<br>GAATGGAGAACGCAG**C**GGGGCGCGATCAAA **(33)**<br>GAATGGAGAACGCAGT**T**GGGCGCGATCAAA **(80)**<br>GAATGGAGAACGCAGTGG**A**GCGCGATCAAA **(27)**<br>GAATGGAGAACGCAGTGGG**T**CGCGATCAAA **(28)**<br>GAATGGAGAACGCAGTGGGG**T**GCGATCAAA **(60)**<br>GAATGGAGAACGCAGTGGGGC**T**CGATCAAA **(221)**	28357-28386

		GAATGGAGAACGCAGTGGGGCCCGATCAAA (158)<br>GAATGGAGAACGCAGTGGGGCACGATCAAA (41)<br>GAATGGAGAACGCAGTGGGGCGCGACCAAA (31)	
	Prb- N	**CATGACGTTCGTGTT**<br>CATGATGTTCGTGTT (31)<br>CATGACGTTTGTGTT (31)<br>CATGACGTTCTTGTT (31)	28227-28241
Department of Pulmonary and Critical Care Medicine	FP-E	**ACTTCTTTTTCTTGCTTTCGTGGT**<br>ACTTCTTTTTGTTGCTTTCGTGGT (125)<br>ACTTCTTTTTTTTGCTTTTGTGGT (33)<br>ACTTCTTTTTCTTGCTTTTGTGGT (285)	26295-26318
	RP-E	**GCAGCAGTACGCACACAATC**<br>GATTGTGTGCGTACTGCTGC	26357-26376
	Prb-E	**CTAGTTACACTAGCCATCCTTACTGC**<br>CTAGTTATACTAGCCATCCTTACTGC (29)	26326-26352
Institute of Medical Microbiology, Virology and Hygiene(UKE)	FP-E	**ACAGGTACGTTAATAGTTAATAGCmGT**<br>ATAGGTACGTTAATAGTTAATAGCGT (196)	26269-26292
	RP-E	**ATATTGCAGCAGTACGCACAmCA**<br>TGTGTGCGTACTGCTGCAATAT	26362-26381
	Prb-E	**ATCCTTACTGCGCTTCG**<br>ATCCTTACTGCGCTTCG	26341-26357
State Key Laboratory of Emerging Infectious Diseases	FP-RdRp	**CGCATACAGTCTTRCAGGCT**	16220-16239
	RP- RdRp	**GTGTGATGTTGAWATGACATGGTC**<br>GACCATGTCATWTCAACATCACAC<br>GATCATGTCATWTCAACATCACAC (40)<br>GACCATGTTATWTCAACATCACAC (41)<br>GACCATGTCATWTCAACATTACAC (65)	16330-16353
	Prb- RdRp	**TTAAGATGTGGTGCTTGCATACGTAGAC**<br>TTAAGATGTGGTGTTTGCATACGTAGAC (41)<br>TTAAGATGTGGTGCTTGTATACGTAGAC (129)	16276-16303
	FP-N	**GCGTTCTTCGGAATGTCG**<br>GCGTTCTTTGGAATGTCG (146)<br>GCGTTCTTCGGAATGTCT (166)	29210-29227
	RP-N	**TTGGATCTTTGTCATCCAATTTG**<br>CAAATTGGATGACAAAGATCCAA<br>TAAATTGGATGACAAAGATCCAA (69)<br>CAAATTGGATGATAAAGATCCAA (159)	29284-29306

		CAAATTGGATGACAAAGA**C**CCAA (34)<br>CAAATTGGATGACAAAGAT**T**CAA (35)	
	Prb-N	**AACGTGGTTGACCTACACAGST**<br>AACGTGGTTGACCTACACAGST<br>AAC**T**TGGTTGACCTACACAGST (142)<br>AAC**C**TGGTTGACCTACACAGST (54)<br>AAC**A**TGGTTGACCTACACAGST (37)<br>AACGTGGTT**A**ACCTACACAGST (548)<br>AACGTGGTTGACCTA**T**ACAGST (377)<br>AACGTGGTTGACCTACA**T**AGST (76)	29257-29278
Zheng Zhou Zhong Dao Biotechnology	FP-RdRp	**TGACACAGACTTTGTGAATGAGTTT**<br>T**T**ACACAGACTTTGTGAATGAGTTT (30)<br>TGA**T**ACAGACTTTGTGAATGAGTTT (102)<br>TGACA**T**AGACTTTGTGAATGAGTTT (58)	15651-15675
	RP- RdRp	**CAGCATCGTCAGAGAGTATCATC**<br>GATGATACTCTCTGACGATGCTG<br>GATGATACT**T**TCTGACGATGCTG (53)<br>GATGATACTCTCTGA**T**GATGCTG (534)	15705-15727
	Prb- RdRp	**ACGCATATTTGCGTAAAC**	15677-15694
Hang zhou Qianji Biotechnology	FP-ORF3a	**TTATACTGAAAAATGGGAATC**<br>TTATACTGAAAAATGGGA**C**TC (38)<br>TTATA**T**TGAAAAATGGGAATC (29)<br>TTATACTGAAAAATGGGAAT**T** (26)<br>TT**G**TACTGAAAAATGGGAATC (26)	25956-25976
	RP-ORF3a	**TAACATGTTCAACACCAGTGTC**<br>GACACTGGTGTTGAACATGTTA<br>GACA**T**TGGTGTTGAACATGTTA (406)<br>GACACT**T**GTGTTGAACATGTTA (355)<br>GACACTGGT**T**TTGAACATGTTA (330)<br>GA**T**ACTGGTGTTGAACATGTTA (298)<br>GACA**A**TGGTGTTGAACATGTTA (86)<br>GACACTG**T**TGTTGAACATGTTA (77)<br>**T**ACACTGGTGTTGAACATGTTA (57)	26056-26077
	Prb-ORF3a	**TACACAGTTACTTCACTTCAGAC**<br>TACACAGTTA**T**TTCACTTCAGAC (26)<br>TACACAGTTACTT**T**ACTTCAGAC (175)<br>TACACAGTTACTTCACTTCA**T**AC (84)<br>TACACAGTTACTTCACTTCAGA**T** (173)	26000-26022
Da An Gene of Sun Yat-sen	FP-nsp3	**TTAAGCGGACACAATCTTGCT**<br>TTAAG**T**GGACACAATCTTGCT (29)<br>TTAAGCGGA**T**ACAATCTTGCT (65)<br>TTAAGCGGACA**T**AATCTTGCT (75)	3578-3596

University		TTAAGCGGACACAACCTTGCT (284)	
	RP-nsp3	**GTTGAATGTCTTCACCTTTGTTAA**<br>TTAACAAAGGTGAAGACATTCAAC<br>TTAATAAAGGTGAAGACATTCAAC (29)<br>TTAACAAATGTGAAGACATTCAAC (587)<br>TTAACAAAGGTGAAGATATTCAAC (69)<br>TTAACAAAGGTGAAGACATTCAAT (123)	3630-3653
	Prb-nsp3	**CACTGTCTTCATGTTGTCGGCCCAAA**<br>TACTGTCTTCATGTTGTCGGCCCAAA (2170)<br>CATTGTCTTCATGTTGTCGGCCCAAA (73)<br>CACTGTCTTCATGTTATCGGCCCAAA (27)<br>CACTGTCTTCATGTTGTAGGCCCAAA (25)<br>CACTGTCTTCATGTTGTTGGCCCAAA (88)<br>CACTGTCTTCATGTTGTCAGCCCAAA (42)	3602-3627
	FP-N	**AAGAAATTCAACTCCAGGCAGC**<br>CAGAAATTCAACTCCAGGCAGC (35)<br>ACGAAATTCAACTCCAGGCAGC (61)<br>AATAAATTCAACTCCAGGCAGC (130)<br>AAGAAATTCAATTCCAGGCAGC (29)<br>AAGAAATTCAACTCCAGGCAGC (231)<br>AAGAAATTCAACTCTAGGCAGC (7100)<br>AAGAAATTCAACTCCATGCAGC (740)	28855-28876
	RP-N	**GCTGGTTCAATCTGTCAAGCAG**<br>CTGCTTGACAGATTGAACCAGC<br>TTGCTTGACAGATTGAACCAGC (32)<br>CTGCTTGATAGATTGAACCAGC (202)<br>CTGCTTGACAGATTGAATCAGC (66)<br>CTGCTTGACAGATTGAACCATC (42)	28940-28961
	Prb-N	**TCACCGCCATTGCCAGCCA**<br>TGGCTGGCAATGGCGGTGA<br>TTGCTGGCAATGGCGGTGA (117)<br>TGACTGGCAATGGCGGTGA (72)<br>TGTCTGGCAATGGCGGTGA (31)<br>TGGTTGGCAATGGCGGTGA (103)<br>TGGCTTGCAATGGCGGTGA (37)<br>TGGCTGTCAATGGCGGTGA (64)<br>TGGCTGGCAACGGCGGTGA (33)<br>TGGCTGGCAATGGCGTTGA (28)	28902-28920
	FP-E	**CTTTCGTGGTATTCTTGCTAGTT**<br>CTTTCGTGGTGTTCTTGCTAGTT (10)	26309-26331
	RP-E	**CACGTTAACAATATTGCAGCA**<br>TGCTGCAATATTGTTAACGTG<br>TGCTGCAATATTGTTAACTTG (26)	26371-26391

	Prb-E	**TAGCCATCCTTACTGCGCTTCGATTG**	26336-26361
Guangdong Pharmaceutical University	FP-ORF1ab	**AGGTTATGGCTGTAGTTGTGATCAACTCCG**<br>AGGTTATGGCTGTAGTTGTGATCAATTCCG **(34)**<br>AGGTTATGGCTGTAGTTGTGATCAACTTCG **(41)**<br>AGGTTATGGCTGTAGTTGTGATCAACTCTG **(167)**	13396-13425
	RP-ORF1ab	**AGTACTAGTGCCTGTGCCGCACGGTGTAAG**<br>CTTACACCGTGCGGCACAGGTACTAGTACT **(44)**<br>CTTACACCGTGCGGCACAGGCATTAGTACT **(433)**	13495-13524
	Prb-ORF1ab	**GAACCCATGCTTCAGTCAGCTGATGCACAA**	13427-13460
Sichuan Academy of Medical Sciences – Sichuan Provicial People's Hospital(SAMSPH)	FP-ORF1ab	**ATGTGTGGCGGTTCACTATATG**	15442-15463
	RP-ORF1ab	**CCGTGACAGCTTGACAAATG**<br>CATTTGTCAAGCTGTCACGG<br>CATTTGTCAAGCCGTCACGG **(29)**<br>CATTTGTCAAGCTGTTACGG **(223)**<br>CATTTGTCAAGCTGTCACTG **(150)**	15525-15544
	Prb-ORF1ab	**CAGGTGGAACCTCATCAGGAGATGC**<br>CAGGTGGAACTTCATCAGGAGATGC **(403)**<br>CAGGTGGAACCTCATCAGGGGATGC **(51)**	15470-15494
	FP-E	**ACAGGTACGTTAATAGTTAATAGCGT**<br>ATAGGTACGTTAATAGTTAATAGCGT **(196)**	26269-26294
	RP-E	**ATATTGCAGCAGTACGCACACA**	26360-26381
	Prb-E	**ACACTAGCCATCCTTACTGCGCTTCG**<br>ATACTAGCCATCCTTACTGCGCTTCG **(29)**	26332-26357
	FP-N	**CTTGAATACACCAAAAGATCACATT**<br>CTTTAATACACCAAAAGATCACATT **(167)**<br>CTTCAATACACCAAAAGATCACATT **(42)**<br>CTTGAATATACCAAAAGATCACATT **(98)**<br>CTTGAATACATCAAAAGATCACATT **(75)**<br>CTTGAATACACCAAAATATCACATT **(57)**<br>CTTGAATACACCAAAACATCACATT **(42)**<br>CTTGAATACACCAAAAAATCACATT **(34)**<br>CTTGAATACACCAAAAGATTACATT **(263)**<br>CTTGAATACACCAAAAGATCATATT **(78)**	28687-28711
	RP-N	**CTCTGCTCCCTTCTGCGTAG**<br>CTACGCAGAAGGGAGCAGAG<br>CTATGCAGAAGGGAGCAGAG **(28)**	28786-28805

		CTACTCAGAAGGGAGCAGAG (41)<br>CTACGTAGAAGGGAGCAGAG (60)<br>CTACGCAGAAGGGAGTAGAG (125)<br>CTACGCAGAAGGGAGCAGGG (32)	
	Prb-N	**ACTTCCTCAAGGAACAACATTGCCA**<br>ATTTCCTCAAGGAACAACATTGCCA (36)<br>ACTTCCCCAAGGAACAACATTGCCA (196)<br>ACTTCCTAAAGGAACAACATTGCCA (47)<br>ACTTCCTCAAGGAACAATATTGCCA (37)<br>ACTTCCTCAAGGAACAACATTGTCA (71)	28753-<br>28777
Sigma	Prb-S5	**AGACTAATTCTCCTCGGCGGGCACG**<br>CGACTAATTCTCCTCGGCGGGCACG (221)<br>GGACTAATTCTCCTCGGCGGGCACG (41)<br>ATACTAATTCTCCTCGGCGGGCACG (1195)<br>ACACTAATTCTCCTCGGCGGGCACG (137)<br>AGATTAATTCTCCTCGGCGGGCACG (71)<br>AGACTAAATCTCCTCGGCGGGCACG (144)<br>AGACTAATTCTCATCGGCGGGCACG (45472)<br>AGACTAATTCTCGTCGGCGGGCACG (276)<br>AGACTAATTCTCCTCGTCGGGCACG (111)<br>AGACTAATTCTCCTCGCCGGGCACG (27)<br>AGACTAATTCTCCTCGGCGTGCACG (172)<br>AGACTAATTCTCCTCGGCGGGTACG (46)	23592-<br>23616
	FP-S5	**CAGGTATATGCGCTAGTTATCAGAC**<br>CTGGTATATGCGCTAGTTATCAGAC (29)<br>CAGGTATATGTGCTAGTTATCAGAC (27)<br>CAGGTATATGCGTTAGTTATCAGAC (36)<br>CAGGTATATGCGCTGGTTATCAGAC (43)<br>CAGGTATATGCGCTACTTATCAGAC (164)<br>CAGGTATATGCGCTAGTTATCGGAC (42)<br>CAGGTATATGCGCTAGTTATCATAC (381)<br>CAGGTATATGCGCTAGTTATCACAC (167)<br>CAGGTATATGCGCTAGTTATCAAAC (25)<br>CAGGTATATGCGCTAGTTATCAGAT (26)	23565-<br>23589
	RP-S5	**CATTGCCTACACTATGTCACTTGG**<br>CCAAGTGACATAGTGTAGGCAATG<br>TCAAGTGACATAGTGTAGGCAATG (204)<br>CATTGCTTACACTATGTCACTTGG (76)<br>CATTGCCTATACTATGTCACTTGG (92)	23638-<br>23661
	FP-S6	**GCAGGTATATGCGCTAGTTATCAG**<br>TCAGGTATATGCGCTAGTTATCAG (27)<br>GCTGGTATATGCGCTAGTTATCAG (29)<br>GCAGGTATATGTGCTAGTTATCAG (27)<br>GCAGGTATATGCGTTAGTTATCAG (36)	23564-<br>23587

		GCAGGTATATGCGCTGGTTATCAG (43)<br>GCAGGTATATGCGCTACTTATCAG (164)<br>GCAGGTATATGCGCTAGTTATCGG (42)<br>GCAGGTATATGCGCTAGTTATCAT (381)<br>GCAGGTATATGCGCTAGTTATCAC (157)<br>GCAGGTATATGCGCTAGTTATCAA (25)	
	RP-S6	**ACACTGGTAGAATTTCTGTGGTAAC**<br>GTTACCACAGAAATTCTACCAGTGT	23726-<br>23750
Xi'an Bioreal-coming BioMed Center	FP-E	**GAGACAGGTACGTTAATAGTTAATAGC**<br>GAGATAGGTACGTTAATAGTTAATAGC (196)	26266-<br>26292
	RP-E	**CAATATTGCAGCAGTACGCACACA**	26360-<br>26383
	Prb-E	**AGTTACACTAGCCATCCTTACTGCGCTTCGA**<br>AGTTATACTAGCCATCCTTACTGCGCTTCGA (29)	26328-<br>26358
	FP-N	**TGGCAATGGCGGTGATG**<br>TTGCAATGGCGGTGATG (37)<br>TGTCAATGGCGGTGATG (64)<br>TGGCAACGGCGGTGATG (33)<br>TGGCAATGGCGTTGATG (28)	28906-<br>28922
	RP-N	**AGCTGGTTCAATCTGTCAAGCA**<br>TGCTTGACAGATTGAACCAGCT<br>TGCTTGATAGATTGAACCAGCT (202)<br>TGCTTGACAGATTGAATCAGCT (66)<br>TGCTTGACAGATTGAACCATCT (42)<br>TGCTTGACAGATTGAACCAGTT (60)	28941-<br>28962
	Prb-N	**TGCTCTTGCTTTGCTGC**<br>TGCTATTGCTTTGCTGC (195)<br>TGCTTTTGCTTTGCTGC (41)<br>TGCTCTTGTTTTGCTGC (13871)<br>TGCTCTTGCTTTGCTGT (32)	28924-<br>28940
Thailand NIH	FP-N	**CGTTTGGTGGACCCTCAGAT**<br>TGTTTGGTGGACCCTCAGAT (43)<br>CTTTTGGTGGACCCTCAGAT (290)<br>CATTTGGTGGACCCTCAGAT (180)<br>CGTTTGGTAGACCCTCAGAT (33)<br>CGTTTGGTGGACCCTCATAT (63)	28320-<br>28339
	RP-N	**CCCCACTGCGTTCTCCATT**<br>AATGGAGAACGCAGTGGGG<br>AATAGAGAACGCAGTGGGG (64)<br>AATCGAGAACGCAGTGGGG (25)<br>AATGGAGAACACAGTGGGG (50)<br>AATGGAGAACGCGGTGGGG (193)<br>AATGGAGAACGCAGTGGGG (47)<br>AATGGAGAACGCAGCGGGG (33)	28358-<br>28376

		AATGGAGAACGCAGT**T**GGG (80)<br>AATGGAGAACGCAGTGG**A**G (27)<br>AATGGAGAACGCAGTGGG**T** (28)	
	Prb-N	**ACCCCGCATTACGTTTGGTGG**<br>A**A**CCCGCATTACGTTTGGTGG (195)<br>A**T**CCCGCATTACGTTTGGTGG (111)<br>AC**C**CCGCATTACGTTTGGTGG (224)<br>ACC**T**CGCATTACGTTTGGTGG (100)<br>ACCC**T**GCATTACGTTTGGTGG (172)<br>ACCCC**T**CATTACGTTTGGTGG (61)<br>ACCCCGCATTA**T**GTTTGGTGG (43)<br>ACCCCGCATTAC**T**TTTGGTGG (290)<br>ACCCCGCATTAC**A**TTTGGTGG (180)<br>ACCCCGCATTACGTTTGGT**A**G (33)	
Shenzhen United Medical Technology	FP-ORF1ab	**CTGAAGAAGAGCAAGAAGAAGATTG**<br>CTGAAGAAGAGCAAGAAGAAGA**C**TG (32)	3177-3201
	RP-ORF1ab	**TCAACAATTGTTTGAATAGTAGTTG**<br>CAACTACTATTCAAACAATTGTTGA (67)<br>**T**AACTACTATTCAAACAATTGTTGA (25)<br>CAA**T**TA**T**TATTCAAACAATTGTTGA (44316)<br>CAACTACTA**C**TCAAACAATTGTTGA (31)	3261-3285
	Prb-ORF1ab	**TCACTGCCGTCTTGTTGACCAACA**<br>TGTTGGTCAACAAGACGGCAGTGA<br>TGTT**A**GTCAACAAGACGGCAGTGA (51)<br>TGTTG**T**TCAACAAGACGGCAGTGA (190)<br>TGTTGGTC**T**ACAAGACGGCAGTGA (37)<br>TGTTGGTCAACAA**T**ACGGCAGTGA (165)<br>TGTTGGTCAACAAGA**T**GGCAGTGA (53)	3226-3249
Chaozhou Kaipu Biochemistry	FP-N	**TCAGCGTTCTTCGGAATGTC**<br>TCAGCGTTCTT**T**GGAATGTC (146)	29207-29226
	RP-N	**CCAATTTGATGGCACCTGTGTAG**<br>CTACACAGGTGCCATCAAATTGG<br>CTA**T**ACAGGTGCCATCAAATTGG (377)<br>CTACA**T**AGGTGCCATCAAATTGG (76)<br>CTACACAGGTGC**T**ATCAAATTGG (78)<br>CTACACAGGTGCCAT**T**AAATTGG (69)	29269-29291
	Prb-N	**CATGGAAGTCACACCTTCGGGAA**<br>CATGGAAGTCA**T**ACCTTCGGGAA (51)<br>CATGGAAGTCACA**T**CTTCGGGAA (34)<br>CATGGAAGTCACAC**T**TTCGGGAA (27)<br>CATGGAAGTCACACC**C**TCGGGAA (39)<br>CATGGAAGTCACACCTT**T**GGGAA (141)<br>CATGGAAGTCACACCTTC**T**GGAA (123)<br>CATGGAAGTCACACCTTC**A**GGAA (66)	29236-29258

The red letters represent the mutated bases. The red number represents the total number of mutation sequences.
