# Supplementary material for: The Impact of Accumulated Mutations in SARS-CoV-2 Variants on the qPCR Detection Efficiency
Source: Front Cell Infect Microbiol. 2022 Jan 28;12:823306. doi: 10.3389/fcimb.2022.823306 (PMC8834649; doi:10.3389/fcimb.2022.823306)
Supplement: Supplementary file 4 [file Table_3.pdf]

Table S3. The deletion mutation of primer/probe sets in the sequence of SARS-CoV-2 (89,791 high-quality SARS-CoV-2 sequences)

Institute	Name	Sequence	Position
China CDC	FP-N	**GGGGAACTTCTCCTGCTAGAAT**<br>GGGGAACTTCTCCTG GAAT (6)<br>GGGGAACTTCTCCTGCT AT (16)	28881–28902
	RP-N	**CAGACATTTTGCTCTCAAGCTG**<br>CAGCTTGAGAGCAAAATGTCTG<br>CAGCTTGAGAGCAAAA CTG (22)	28958–28979
	Prb-N	**TTGCTGCTGCTTGACAGATT**	28934–28953
HKU CDC	FP-nsp14	**TGGGGYTTTACRGGTAACCT**	18778–18797
	RP-nsp14	**AACRCGCTTAACAAAGCACTC**<br>GAGTGCTTTGTTAAGCGTGTT<br>GAGTGCT TTAAGCGTGTT (10)	18889–18909
	Prb-nsp14	**TAGTTGTGATGCWATCATGACTAG**	18849–18872
Northwell Health Laboratories	FP-S	**TCAACTCAGGACTTGTTCTTAC**	21710-21731
	RP-S	**TGGTAGGACAGGGTTATCAAAC**<br>GTTTGATAACCCTGTCCTACCA	21796-21817
	Prb-S	**TGGTCCCAGAGACATGTATAGCAT**<br>ATGCTATACATGTCTCTGGGACCA<br>ATGCT GTCTCTAGGACCA (12)<br>ATGCTA TCTCTAGGACCA (32749)<br>ATGCTAT GTCTCTAGGACCA (5)	21759-21782
Charité	FP-E	**ACAGGTACGTTAATAGTTAATAGCGT**	26269-26294
	RP-E	**ATATTGCAGCAGTACGCACACA**<br>TGTGTGCGTACTGCTGCAATAT	26360-26381
	Prb-E	**ACACTAGCCATCCTTACTGCGCTTCG**<br>ATACTAGCCATCCTTAC TCG (3)<br>ATACTAGCCATCCTTACTG G (3)	26332-26357
University of Malaya	FP-N	**TTGTTCGTTCTATGAAGACTTTTTAGAG**	28196-28223
	RP- N	**TTTGATCGCGCCCCACTGCGTTCTCCATTC**<br>GAATGGAGAACGCAGTGGGGCGCGATCAAA	28357-28386

	Prb- N	**CATGACGTTCGTGTT**<br>CATGACGTTCG T (8)	28196-28225
Institute of Microbiology and Virology	FP-N	**CCTCTTCTCGTTCCTCATCACGTAGTCGCAAC**<br>CCTATTCTCGTTCCTCATCACGT (10)	28818-28849
	RP-N	**AGTGACAGTTTGGCCTTGTTGTTGTTGGCCTT**<br>AAGGCCAACAACAACAAGGCCAAACTGTCACT	28926-28960
	Prb-N	**CCTGCTAGAATGGCTGGCAATGGCGGTGA**	28880-28908

The red number represents the total number of mutation sequences.
