# Supplementary material for: The Impact of Accumulated Mutations in SARS-CoV-2 Variants on the qPCR Detection Efficiency
Source: Front Cell Infect Microbiol. 2022 Jan 28;12:823306. doi: 10.3389/fcimb.2022.823306 (PMC8834649; doi:10.3389/fcimb.2022.823306)
Supplement: Supplementary file 5 [file Table_4.pdf]

Table S4. The point mutation of primer/probe sets in SARS-CoV-2 Variants

**92,214 high quality SARS-CoV-2 Alpha variant**			
Institute	Name	Sequence	Position
China CDC	FP-N	**GGGGAACTTCTCCTGCTAGAAT**<br>AACGAACTTCTCCTGCTAGAAT (79605)<br>AACCAACTTCTCCTGCTAGAAT (11853)	28881–28902
	RP-N	**CAGACATTTTGCTCTCAAGCTG**<br>CAGCTTGAGAGCAAAATGTCTG<br>CAGCTTGAGAGCAAAATATTTG (91904)	28958–28979
	Prb-N	**TTGCTGCTGCTTGACAGATT**	28934–28953
University of Leipzig	FP-RdRp	**TATGCCATTAGTGCAAAGAATAGAGCTCGCAC**<br>TATGCCATTAGTGCAAAGAACAGAGCTCGCAC (27213)	15076-15107
	RP-RdRp	**CAACCACCATAGAATTTGCTTGTTCCAATTAC**<br>GTAATTGGAACAAGCAAATTCTATGGTGGTTG	15202-15233
	Prb-RdRp	**TCCTCTAGTGGCGGCTATTGATTTCAATAA**<br>TTATTGAAATCAATAGCCGCCACTAGAGGA	15163-15192
sigma	FP-S5	**CAGGTATATGCGCTAGTTATCAGAC**	23565-23589
	RP-S5	**CCAAGTGACATAGTGTAGGCAATG**<br>CATTGCCTACACTATGTCACTTGG	23638-23661
	Prb-S5	**AGACTAATTCTCCTCGGCGGGCACG**<br>AGACTAATTCTCATCGGCGGGCACG (91682)	23592-23616
	FP-S6	**GCAGGTATATGCGCTAGTTATCAG**	23564-23587
	RP-S6	**ACACTGGTAGAATTTCTGTGGTAAC**	23726-23750
Shenzhen United Medical Technology Co., Ltd	FP-ORF1ab	**CTGAAGAAGAGCAAGAAGAAGATTG**	3177-3201
	RP-ORF1ab	**TCAACAATTGTTTGAATAGTAGTTG**<br>CAACTACTATTCAAACAATTGTTGA<br>CAAATATTATTCAAACAATTGTTGA (92122)	3261-3285
	Prb-ORF1ab	**TCACTGCCGTCTTGTTGACCAACA**<br>TGTTGGTCAACAAGACGGCAGTGA	3226-3249
**6,341 high-quality SARS-CoV-2 Beta variants**			
Institute	Name	Sequence	Position
China CDC	FP-N	**GGGGAACTTCTCCTGCTAGAAT**<br>GGGGAATTTCTCCTGCTAGAAT (6033)	28881–28902

	RP-N	**CAGACATTTTGCTCTCAAGCTG**<br>CAGCTTGAGAGCAAAATGTCTG	28958–28979
	Prb-N	**TTGCTGCTGCTTGACAGATT**	28934–28953
Northwell Health Laboratories	FP-S	**TCAACTCAGGACTTGTTCTTAC**	21710-21731
	RP-S	**TGGTAGGACAGGGTTATCAAAC**<br>GTTTGATAACCCTGTCCTACCA<br>GTTTG**C**TAACCCTGTCCTACCA **(6094)**	21796-21817
	Prb-S	**TGGTCCCAGAGACATGTATAGCAT**<br>ATGCTATACATGTCTCTGGGACCA	21759-21782
State Key Laboratory of Emerging Infectious Diseases	FP-S	**CCTACTAAATTAAATGATCTCTGCTTTACT**	22712-22741
	RP-S	**CAAGCTATAACGCAGCCTGTA**<br>TACAGGCTGCGTTATAGCTTG	22849-22869
	Prb-S	**CGCTCCAGGGCAAACTGGAAAG**<br>CGCTCCAGGGCAAACTGGAAA**T** **(5877)**	22792-22813
**13,655 high-quality SARS-CoV-2 Gamma variants**			
Institute	Name	Sequence	Position
China CDC	FP-N	**GGGGAACTTCTCCTGCTAGAAT**<br>**AAC**GAACTTCTCCTGCTAGAAT **(13587)**	28881–28902
	RP-N	**CAGACATTTTGCTCTCAAGCTG**<br>CAGCTTGAGAGCAAAATGTCTG	28958–28979
	Prb- N	**TTGCTGCTGCTTGACAGATT**	28934–28953
State Key Laboratory of Emerging Infectious Diseases	FP-S	**CCTACTAAATTAAATGATCTCTGCTTTACT**	22712-22741
	RP-S	**CAAGCTATAACGCAGCCTGTA**<br>TACAGGCTGCGTTATAGCTTG	22849-22869
	Prb-S	**CGCTCCAGGGCAAACTGGAAAG**<br>CGCTCCAGGGCAAACTGGAA**C**G **(13609)**	22792-22813
**211,740 high-quality SARS-CoV-2 Delta variants**			

Institute	Name	Sequence	Position
China CDC	FP-N	**GGGGAACTTCTCCTGCTAGAAT**<br>TGGGAACTTCTCCTGCTAGAAT (211381)	28881–28902
	RP-N	**CAGACATTTTGCTCTCAAGCTG**<br>CAGCTTGAGAGCAAAATGTCTG	28958–28979
	Prb-N	**TTGCTGCTGCTTGACAGATT**	28934–28953
Institute of Microbiology and Virology	FP-N	**CCTCTTCTCGTTCCTCATCACGTAGTCGCAAC**	28818-28849
	RP-N	**AGTGACAGTTTGGCCTTGTTGTTGTTGGCCTT**<br>AAGGCCAACAACAACAAGGCCAAACTGTCACT	28983-29014
	Prb-N	**CCTGCTAGAATGGCTGGCAATGGCGGTGA**<br>TCTGCTAGAATGGCTGGCAATGGCGGTGA<br>TCTGCTAGAATGGCTGGCAATGGCTGTGA (166809)	28892-28920
Da An Gene of Sun Yat-sen University	FP-N	**AAGAAATTCAACTCCAGGCAGC**	28855-28876
	RP-N	**GCTGGTTCAATCTGTCAAGCAG**<br>CTGCTTGACAGATTGAACCAGC	28940-28961
	Prb-N	**TCACCGCCATTGCCAGCCA**<br>TGGCTGGCAATGGCGGTGA<br>TGGCTGGCAATGGCTGTGA (166809)	28902-28920
Sichuan Academy of Medical Sciences – Sichuan Provicial People's Hospital(SAMSPH)	FP-ORF1ab	**ATGTGTGGCGGTTCACTATATG**<br>ATGTGTGGCAGTTCACTATATG (206813)	15442-15463
	RP-ORF1ab	**CCGTGACAGCTTGACAAATG**<br>CATTTGTCAAGCTGTCACGG	15525-15544
	Prb-ORF1ab	**CAGGTGGAACCTCATCAGGAGATGC**	15470-15494
sigma	FP-S5	**CAGGTATATGCGCTAGTTATCAGAC**	23565-23589
	RP-S5	**CCAAGTGACATAGTGTAGGCAATG**<br>CATTGCCTACACTATGTCACTTGG	23638-23661
	Prb-S5	**AGACTAATTCTCCTCGGCGGGCACG**<br>AGACTAATTCTCGTCGGCGGGCACG (211272)	23592-23616
Xi'an Bioreal-coming BioMed Center	FP-N	**TGGCAATGGCGGTGATG**<br>TGGCAATGGCTGTGATG (166809)	28906-28922
	RP-N	**AGCTGGTTCAATCTGTCAAGCA**<br>TGCTTGACAGATTGAACCAGCT	28941-28962

	Prb-N	**TGCTCTTGCTTTGCTGC**	28924-28940
**2,333 high-quality SARS-CoV-2 Omicron variants**			
Institute	Name	Sequence	Position
China CDC	FP-N	**GGGGAACTTCTCCTGCTAGAAT**<br>**AAC**GAACTTCTCCTGCTAGAAT **(2317)**	28881–28902
	RP-N	**CAGACATTTTGCTCTCAAGCTG**<br>CAGCTTGAGAGCAAAATGTCTG	28958–28979
	Prb-N	**TTGCTGCTGCTTGACAGATT**	28934–28953
USCDC	FP-N1	**GACCCCAAAATCAGCGAAAT**	28287–28306
	RP-N1	**TCTGGTTACTGCCAGTTGAATCTG**<br>CATATTCAACTGGCAGTAACCAGA	28335–28358
	Prb-N1	**ACCCCGCATTACGTTTGGTGGACC**<br>AC**T**CCGCATTACGTTTGGTGGACC **(2297)**	28309–28332
Charité	FP-E	**ACAGGTACGTTAATAGTTAATAGCGT**<br>A**T**AGGTACGTTAATAGTTAATAGCGT **(2312)**	26269-26294
	RP-E	**ATATTGCAGCAGTACGCACACA**<br>TGTGTGCGTACTGCTGCAATAT	26360-26381
	Prb-E	**ACACTAGCCATCCTTACTGCGCTTCG**	26332-26357
Northwell Health Laboratories	FP-S	**TCAACTCAGGACTTGTTCTTAC**	21710-21731
	RP-S	**TGGTAGGACAGGGTTATCAAAC**<br>GTTTGATAACCCTGTCCTACCA	21796-21817
	Prb-S	**TGGTCCCAGAGACATGTATAGCAT**<br>ATGCTATACATGTCTCTGGGACCA<br>ATG**T**TATACATGTCTCTGGGACCA **(2297)**	21759-21782
State Key Laboratory of Emerging Infectious Diseases	FP-S	**CCTACTAAATTAAATGATCTCTGCTTTACT**	22712-22741
	RP-S	**CAAGCTATAACGCAGCCTGTA**<br>TACAGGCTGCGTTATAGCTTG	22849-22869
	Prb-S	**CGCTCCAGGGCAAACTGGAAAG**<br>CGCTCCAGGGCAAACTGGAAA**T** **(1989)**	22792-22813
Sichuan Academy of Medical Sciences –	FP-E	**ACAGGTACGTTAATAGTTAATAGCGT**<br>A**T**AGGTACGTTAATAGTTAATAGCGT**(2312)**	26269-26294
	RP-E	**ATATTGCAGCAGTACGCACACA**	26360-26381

Sichuan Provicial People's Hospital(SAMSPH)	Prb-E	**ACACTAGCCATCCTTACTGCGCTTCG**	26332-26357
Sigma	Prb-S5	**AGACTAATTCTCCTCGGCGGGCACG**<br>AGACTAAGTCTCCTCGGCGGGCACG (2315)<br>AGACTAATTCTCATCGGCGGGCACG (2312)	23592-23616
	FP-S5	**CAGGTATATGCGCTAGTTATCAGAC**	23565-23589
	RP-S5	**CATTGCCTACACTATGTCACTTGG**<br>CCAAGTGACATAGTGTAGGCAATG	23638-23661
Xi'an Bioreal-coming BioMed Center.	FP-E	**GAGACAGGTACGTTAATAGTTAATAGC**<br>GAGATAGGTACGTTAATAGTTAATAGC(2312)	26266-26292
	RP-E	**CAATATTGCAGCAGTACGCACACA**	26360-26383
	Prb-E	**AGTTACACTAGCCATCCTTACTGCGCTTCGA**	26328-26358
**345 high-quality SARS-CoV-2 Lambda variants**			
Institute	Name	Sequence	Position
China CDC	FP-N	**GGGGAACTTCTCCTGCTAGAAT**<br>AACGAACTTCTCCTGCTAGAAT (331)	28881–28902
	RP-N	**CAGACATTTTGCTCTCAAGCTG**<br>CAGCTTGAGAGCAAAATGTCTG	28958–28979
	Prb-N	**TTGCTGCTGCTTGACAGATT**	28934–28953
Da An Gene of Sun Yat-sen University	FP-N	**AAGAAATTCAACTCCAGGCAGC**	28855-28876
	RP-N	**GCTGGTTCAATCTGTCAAGCAG**<br>CTGCTTGACAGATTGAACCAGC	28940-28961
	Prb-N	**TCACCGCCATTGCCAGCCA**<br>TGGCTGGCAATGGCGGTGA<br>TGGCTGGCAATTGCGGTGA (333)	28902-28920
Institute of Microbiology and Virology	FP-N	**CCTCTTCTCGTTCCTCATCACGTAGTCGCAAC**<br>CCTCTTCTCGTTCCTCATCACGTAGTCGCAAT (91)	28818-28849
	RP-N	**AGTGACAGTTTGGCCTTGTTGTTGTTGGCCTT**<br>AAGGCCAACAACAACAAGGCCAAACTGTCACT	28983-29014
	Prb-N	**CCTGCTAGAATGGCTGGCAATGGCGGTGA**<br>CCTGCTAGAATGGCTGGCAATTGCGGTGA (333)	28892-28920
**2,276 high-quality SARS-CoV-2 Mu variants**			
Institute	Name	Sequence	Position

China CDC	N-F	**GGGGAACTTCTCCTGCTAGAAT**<br>GGGGAA**T**TTCTCCTGCTAGAAT **(2270)**	28881–28902
	N-R	**CAGACATTTTGCTCTCAAGCTG**<br>CAGCTTGAGAGCAAAATGTCTG	28958–28979
	N-P	**TTGCTGCTGCTTGACAGATT**	28934–28953
sigma	Prb-S5	**CAGGTATATGCGCTAGTTATCAGAC**	23565-23589
	FP-S5	**CCAAGTGACATAGTGTAGGCAATG**<br>CATTGCCTACACTATGTCACTTGG	23638-23661
	RP-S5	**AGACTAATTCTCCTCGGCGGGCACG**<br>CGACTAATTCTC**A**TCGGCGGGCACG **(2270)**	23592-23616
	FP-S6	**GCAGGTATATGCGCTAGTTATCAG**	23564-23587
	RP-S6	**ACACTGGTAGAATTTCTGTGGTAAC**	23726-23750

The red letters represent the mutated bases. The red number represents the total number of mutation sequences.
